# *EPHA2* Is Associated with Age-Related Cortical Cataract in Mice and Humans

**DOI:** 10.1371/journal.pgen.1000584

**Published:** 2009-07-31

**Authors:** Gyungah Jun, Hong Guo, Barbara E. K. Klein, Ronald Klein, Jie Jin Wang, Paul Mitchell, Hui Miao, Kristine E. Lee, Tripti Joshi, Matthias Buck, Preeti Chugha, David Bardenstein, Alison P. Klein, Joan E. Bailey-Wilson, Xiaohua Gong, Tim D. Spector, Toby Andrew, Christopher J. Hammond, Robert C. Elston, Sudha K. Iyengar, Bingcheng Wang

**Affiliations:** 1 Department of Epidemiology and Biostatistics, Case Western Reserve University School of Medicine, Cleveland, Ohio, United States of America; 2 Rammelkamp Center for Research, Department of Pharmacology and Ireland Comprehensive Cancer Center, Case Western Reserve University School of Medicine, Cleveland, Ohio, United States of America; 3 Department of Ophthalmology and Visual Sciences, University of Wisconsin School of Medicine and Public Health, Madison, Wisconsin, United States of America; 4 Centre for Vision Research, Westmead Millennium Institute, Westmead Hospital, Department of Ophthalmology, University of Sydney, Sydney, Australia; 5 Centre for Eye Research Australia and Department of Ophthalmology, University of Melbourne, Melbourne, Australia; 6 Department of Physiology and Biophysics, Case Western Reserve University School of Medicine, Cleveland, Ohio, United States of America; 7 Ocular Oncology Service, Department of Ophthalmology and Pathology, Case Western Reserve University School of Medicine, Cleveland, Ohio, United States of America; 8 Sidney Kimmel Comprehensive Cancer Center, Johns Hopkins University School of Medicine, Baltimore, Maryland, United States of America; 9 Inherited Disease Research Branch, National Human Genome Research Institute, National Institutes of Health, Baltimore, Maryland, United States of America; 10 School of Optometry and Vision Science Program, University of California Berkeley/University of California San Francisco Joint Bioengineering Graduate Program, University of California Berkeley, Berkeley, California, United States of America; 11 King's College London, St Thomas' Hospital Campus, Twin Research and Genetic Epidemiology Unit, London, United Kingdom; 12 Department of Genetics, Case Western Reserve University School of Medicine, Cleveland, Ohio, United States of America; 13 Department of Ophthalmology, Case Western Reserve University School of Medicine, Cleveland, Ohio, United States of America; Medical Research Council Human Genetics Unit, United Kingdom

## Abstract

Age-related cataract is a major cause of blindness worldwide, and cortical cataract is the second most prevalent type of age-related cataract. Although a significant fraction of age-related cataract is heritable, the genetic basis remains to be elucidated. We report that homozygous deletion of *Epha2* in two independent strains of mice developed progressive cortical cataract. Retroillumination revealed development of cortical vacuoles at one month of age; visible cataract appeared around three months, which progressed to mature cataract by six months. EPHA2 protein expression in the lens is spatially and temporally regulated. It is low in anterior epithelial cells, upregulated as the cells enter differentiation at the equator, strongly expressed in the cortical fiber cells, but absent in the nuclei. Deletion of *Epha2* caused a significant increase in the expression of HSP25 (murine homologue of human HSP27) before the onset of cataract. The overexpressed HSP25 was in an underphosphorylated form, indicating excessive cellular stress and protein misfolding. The orthologous human *EPHA2* gene on chromosome 1p36 was tested in three independent worldwide Caucasian populations for allelic association with cortical cataract. Common variants in *EPHA2* were found that showed significant association with cortical cataract, and rs6678616 was the most significant in meta-analyses. In addition, we sequenced exons of *EPHA2* in linked families and identified a new missense mutation, *Arg721Gln*, in the protein kinase domain that significantly alters EPHA2 functions in cellular and biochemical assays. Thus, converging evidence from humans and mice suggests that *EPHA2* is important in maintaining lens clarity with age.

## Author Summary

Cataract is the leading cause of blindness. Cataract may form at any age, but the peak incidence is bimodal—in the perinatal period or later than 50 years of age. The early onset forms follow Mendelian inheritance patterns and are rare. Age-related cataract accounts for 18 million cases of blindness and 59 million cases of reduced vision worldwide. Among three types of age-related cataract, cortical cataract is known to be highly heritable, although few genes have been linked to its etiology. We report here that *EPHA2* is associated with cortical cataract. *EPHA2* is expressed in mouse and human cortical lens fiber cells, and homozygous deletion of *Epha2* in two independent strains of mice led to development of cataract that progressed with age. Common and rare variants including a missense mutation in the *EPHA2* gene were associated for cortical cataract in three different Caucasian populations. Our study identified *EPHA2* as a gene for human age-related cataract and established *Epha2* knockout mice as a model for progressive cortical cataract.

## Introduction

Cataract is the leading cause of visual impairment worldwide, with approximately 37 million people affected, accounting for 48% of global blindness [1]. Cataract is defined as any opacity of the crystalline lens resulting from either alteration in lens cell structure or changes in lens proteins or both. Morphologic classification of age-related cataract can be divided into four categories: nuclear, cortical, posterior subcapsular cataract and mixed type. Among Caucasian Americans aged 65 years or older, the reported prevalence of cortical cataract was 24% [2]. Incidence of cataract surgery in the United States is highest amongst those over 70 years, with an annual increase of about 14% [3].

Age-related cataract has consistently been attributed to risk factors such as old age, female gender, diabetes, hypertension, smoking, UV light exposure, and heavy alcohol intake [4]. Recent studies showed excess clustering of disease in families [4]. Heritability for cortical cataract in female twins was estimated as high as 58%, and at least 11% of total variance was accounted for by age [5]. In segregation analysis in the Beaver Dam Eye Study (BDES), a single major gene accounted for 58% of the variability of age- and gender-adjusted measures of cortical cataract [6]. Genes involved in the oxidative stress pathway [7], hypergalactosemia [8], hyperferritinemia [9], diabetic complications [10], and neurodegenerative disease [11],[12], mediate development of juvenile or age-related cataract.

A genome-wide scan for cortical cataract after adjusting for covariates in a subset of the BDES showed evidence of linkage in 1p36 [13]. This region is one of the most replicated loci for inherited cataract including the Volkman (pulverulent) cataract from a large Danish pedigree [14], a type of posterior polar cataract [15], and a total congenital cataract from a six-generation Australian family [16]. The Eph receptor A2 gene (*EPHA2*) that resides under the linkage peak on 1p36 was selected as a candidate gene because knockout mice for *Epha2* developed age-related cortical cataract. In this study, we extensively examined characteristics of the EPHA2 protein using knockout mice. We investigated allelic association by re-sequencing and genotyping of single nucleotide polymorphisms (SNPs) in coding and non-coding conserved regions in large population-based human studies.

## Results

### *Epha2* Knockout Mice Developed Progressive Cortical Cataract

**Figure 1:**
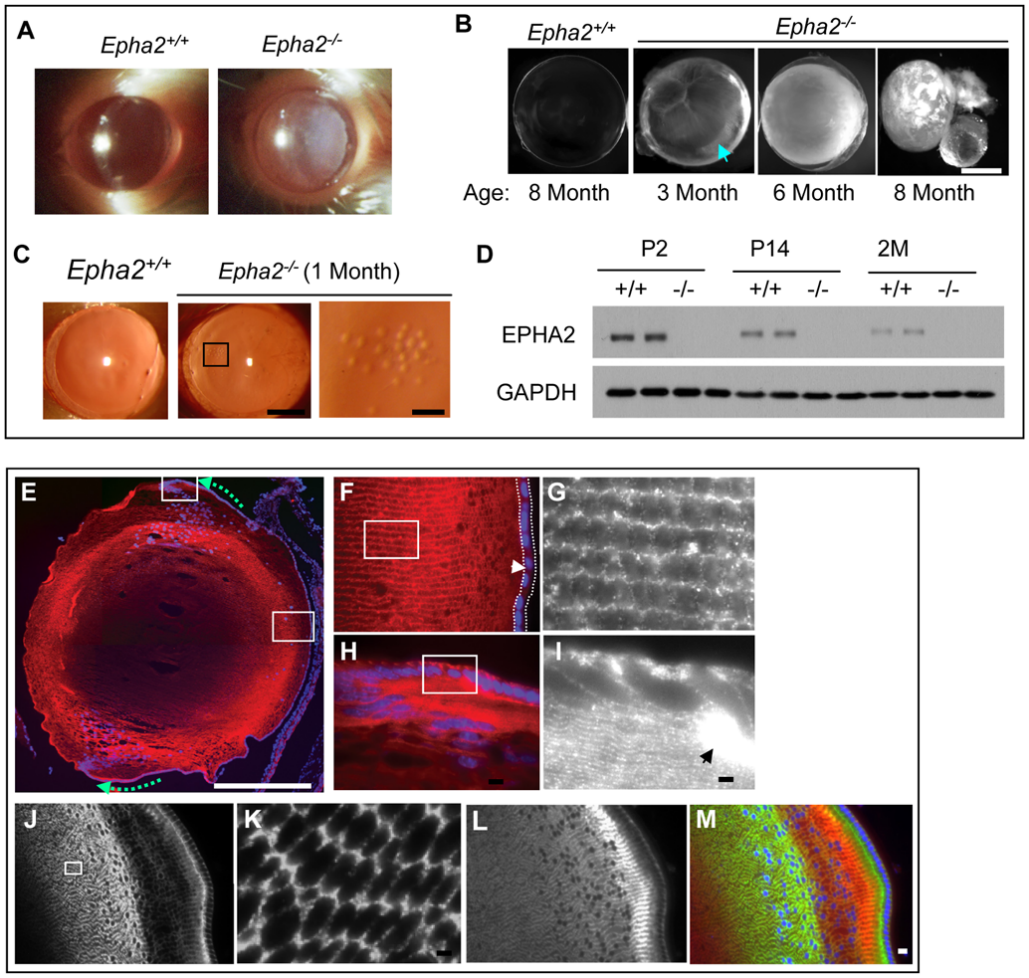
Development of progressive cataract in *Epha2* knockout mice and characterization of spatial and temporal regulation of EPHA2 expression in the lens. (A–C) *Epha2* homozygous deletion in mice causes development of progressive cataract. (A) Cataracts were visible by gross inspection in homozygous *Epha2* knockout mice (*Epha2^−/−^*) between 5 to 8 months of age, but not in heterozygous or wild type mice. Shown are slit lamp images confirming development of cataract in *Epha2^−/−^* but not *Epha2^+/+^* mice. (B) Dark field imaging of the dissected lens. Although not readily detectable by visual inspection, cataracts were found on dissected lens by 3 months of age. This lens was tilted to show denser opacity near the equator (arrow). Enucleation frequently occurred during dissection of mature cataract after 8 months (far right). (C) Retroillumination examination revealed clusters of small vacuoles by one month of age. Scale bars: 1 mm for middle panel; 150 µm for right panel. (D) Immunoblot of total lens lysates showing decreasing EPHA2 expression with aging. (E–M) Compartmentalized and gradient expression of EPHA2 (red) in mouse lens. Blue: DAPI nuclear staining. (E–I) Midsagittal sections of lens from 14-day-old wild type mice were stained for EPHA2. (E) Low power view of an entire lens revealed dense expression of EPHA2 in subcortical lens fiber cells. Dotted arrows indicate gradient expression in lens epithelial cells near the equator. Scale bar: 1 mm. (F) Low EPHA2 expression in anterior lens epithelial cells (arrow head, sandwiched between dotted lines). (G) Inset from (F) showing high EPHA2 expression in lens fiber cells. (H) High level of EPHA2 expression at the bow. (I) Inset from (H) showing dense expression at modulus (arrow). Scale bars: 5 µm for F–I. (J–M) Coronal sections through the bow region of lens co-stained for EPHA2 and N-cadherin. (J) Note the spatially regulated expression pattern in subcortical lens fiber cells. (K) Inset from (J) showing “honey-comb” membrane staining pattern of EPHA2 in the cross sections of fiber cells at high magnifications. (L) N-cadherin from the same section show overlapping but distinct expression pattern compared with that of EPHA2. (M) Merged images of EPHA2/N-cadherin. 10 µm for J, L, and M; 2 µm for K.

During the course of characterizing mice with deletion of *Epha2* gene, we observed development of progressive cortical cataract in a significant fraction of homozygous knockout mice. This line of knockout mice (*Epha2^−/−^*) with deletion of exons 6–17 was generated using a secretory gene trapping strategy [17],[18]. Initially noticed on FVB/NJ genetic background, visible bilateral lens opacity in *Epha2^−/−^* mice appeared by five months, which was confirmed as cataract by slit lamp examination (Figure 1A). The incidence and severity increased with age, affecting over 80% of mice by 12 months (Table 1). Inspection of dissected lens revealed significant lens opacity between three to four months, and mature cataracts with lens rupture occurred between six to eight months (Figure 1B and Figure S1A). Dense opacity initiated around the equator and progressed to involve the entire lens (Figure 1B). As early as one month after birth, prior to the onset of lens opacity, retroillumination examination revealed clusters of subcapsular vacuoles in the anterior cortex of the *Epha2* knockout but not in heterozygous or wild-type lenses (Figure 1C), which are indicative of structural and osmotic alterations in the lens [19].

**Table 1:** Incidence of Visible Cataracts in Wild-Type and *Epha2*-null Mice.

Age of animal	Equivalent human age	Incidence of cataracts (%)		
		+/+	+/−	−/−
2 months	5 years	0 (0/6)	0 (0/6)	0 (0/6)
5 months	18 years	0 (0/9)	0 (0/15)	26.1 (6/23)
6 months	20 years	0 (0/10)	0 (0/15)	52.6 (10/19)
8 months	28 years	0 (0/18)	0 (0/27)	73.5 (36/49)
14 months	40 years	0 (0/14)	0 (0/23)	83.3 (15/18)

Note: Cataract development was determined by visual inspection every two weeks. Microscopic examination of the dissected lens showed cataract occurred as early as three months of age (see Figure 1B).

To further confirm a link between loss of *Epha2* gene and cataractogenesis, we examined another *Epha2* mutant mouse line on C57Bl/6 genetic background, where a retrovirus is inserted in the first intron, inactivating expression of *Epha2* [20]. The homozygous mutant mice developed cataracts with similar morphological changes as the secretory gene trapping mice on FVB/NJ background described above. Thus, neither the strain genetic backgrounds nor the secretory trapping of partial EPHA2 ectodomain fused to β-gal is likely to be a major contributing factor in the lens phenotypes of the *Epha2* mutant mice. Interestingly, in cohorts of mice subject to two-stage chemical skin carcinogenesis studies [17], the penetrance of cataract increased to 100% by six months, suggesting that similar to humans, environmental factors could significantly influence cataractogenesis in *Epha2*-null mice.

### EPHA2 Protein Is Expressed in Mice and Human Lens

We found that EPHA2 was expressed in lens homogenates of wild type mice, and the level of expression progressively decreased with age (Figure 1D). The relative level of expression is compatible with other tissues known to have high levels of EPHA2 such as the skin [17]. Immunofluorescence analysis revealed that EPHA2 was most abundantly expressed in cortical lens fiber cells but not nuclear fiber cells (Figure 1E). In lens epithelial cells, EPHA2 expression was low in the anterior region and became upregulated when epithelial cells underwent differentiation at the lens equator (Figure 1E–1K). Much weaker staining was seen in the nuclei of the lens. To verify these findings, we separated lens cortex and nuclei from two week and five month old mice and subjected the homogenates to immunoblot. EPHA2 was highly expressed in cortical fiber cells at two weeks, which fell significantly by five months (Figure S1F). Histochemical staining for LacZ reporter gene cassette confirmed the elevated EPHA2 expression near the equator (Figure S1XPATH ERROR: unknown variable "checknextn".). Consistent with our observations in the mouse, EPHA2 was readily detectable in human lens fiber cells by immunoblot and immunohistochemistry (Figure S2).

**Figure 2:**
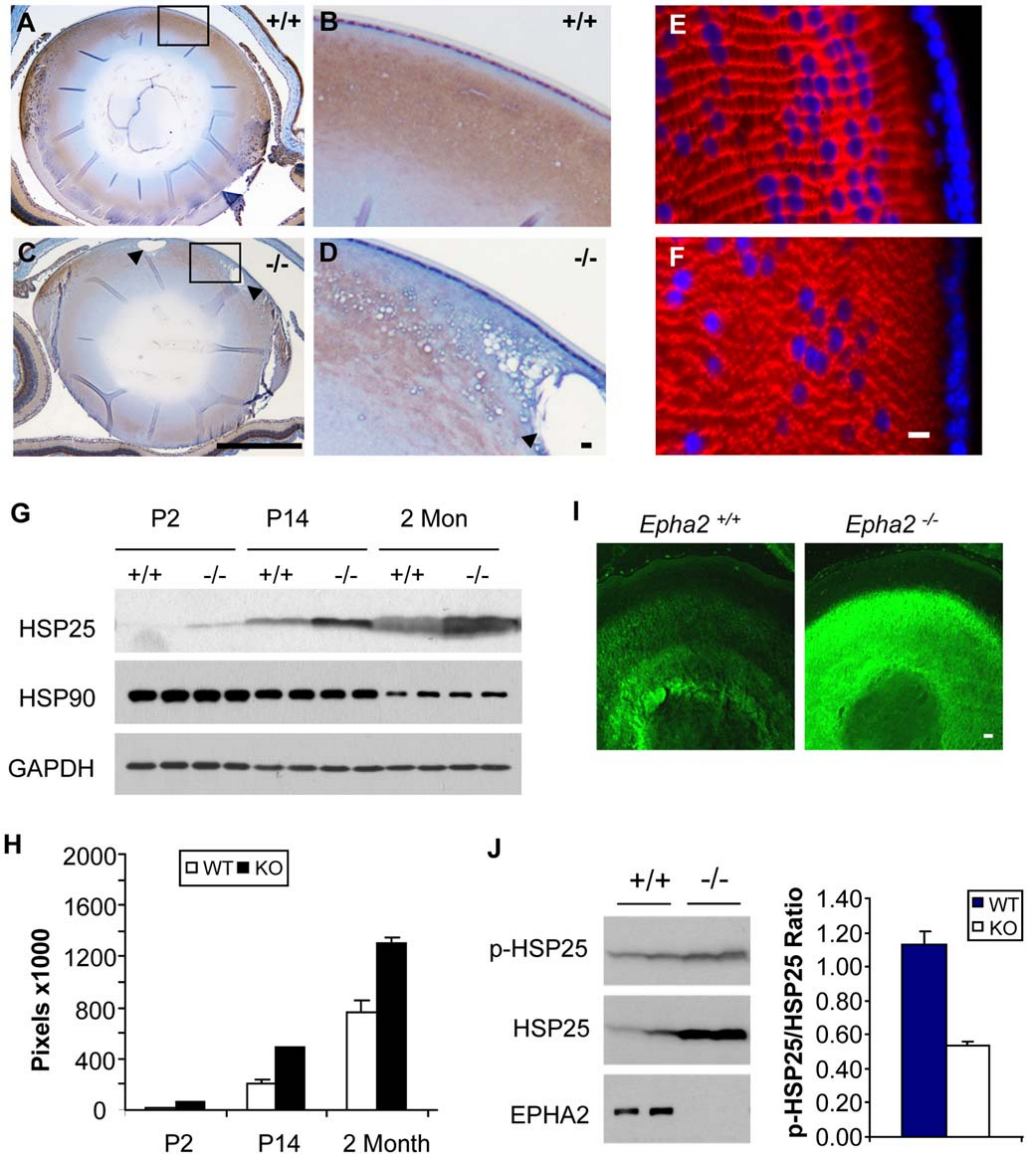
Structural alteration and stress responses in *Epha2^−/−^* lens. (A–D) Expression of ephrin-A1, a ligand for EPHA2. Note the disorganized ephrin-A1 expression and formation of vacuoles in the *Epha2^−/−^* lens (arrow heads). Scale bars: 1 mm for A and C; 10 µm for B and D. (E,F) N-cadherin staining showing disorganization of lens fiber cells. Scale bars: 5 µm. (G) Overexpression of HSP25 but not HSP90 in *Epha2^−/−^* lens which was quantified in (H), and confirmed by immunofluorescence staining (I). Scale bars: 40 µm. (J) Immunoblot for phosphorylated HSP25 revealed relatively low degree of phosphorylation in *Epha2^−/−^* lens.

Eph kinases bind to membrane-anchored ligands called ephrins and mediate cell contact-dependent bidirectional signaling [21],[22]. We found that ephrin-A1, a ligand for EPHA2, displayed a similar expression pattern with strong expression in lens fiber cells as well as lens epithelial cells near the equator (Figure 2A and 2B). The expression of EPHA2 and ephrin-A1 in the overlapping regions suggests EPHA2-ephrin-A1 interaction at these sites, which could have signaling and/or structural roles in the lens. Homozygous deletion of *Epha2* gene led to significant alteration in ephrin-A1 localization (Figure 2C and 2D) concomitant with changes in lens structures (Figure 2D). Staining for expression of N-cadherin (Figure 1L and 1M), a known component of cell adhesion junctions in lens fiber cells, further confirmed structural defects in the mutant lens (Figure 2E and 2F).

### Overexpression of HSP25 in *Epha2* ^−/−^ Mouse Lens

Disruption of lens structure could be caused by cellular stresses. We found that HSP25, a small heat shock protein and the murine homologue of human HSP27, was significantly overexpressed in the *Epha2* knockout lens from day two after birth, long before the onset of cataract (Figure 2G–2I). Expression of HSP90, on the other hand, was not affected. Prior to the onset of lens degeneration at old age (6 months and beyond), expression of major crystallins did not show significant change either (Figure S3A). HSP25 is closely related to and directly interacts with αB-crystallin, being expressed during oxidative stress in human cataractous lenses [21]. The overexpressed HSP25 in mutant lenses is in an underphosphorylated form that is known to form large aggregates with misfolded proteins (Figure 2J) [23],[24]. By trapping misfolded proteins in large aggregates, HSP25 could contribute to cataractogenesis. While the exact mechanisms of cataractogenesis remain to be fully elucidated, these results together suggest that *Epha2* deletion probably causes elevated stress responses in the lens, which may lead to increased protein misfolding, cellular structural damages and eventual lens opacity.

### Identification of Coding Variants in Humans Using Re-Sequencing

The human *EPHA2* gene is located on 1p36 where we and others had previously reported linkage with cortical and progressive juvenile onset cataract, respectively [13]–[16]. This, together with the *Epha2* knockout mouse data described above, motivated us to investigate association between cortical cataract and *EPHA2* in humans. For discovery, we used a population-based family sample from the US Beaver Dam Eye Study (BDES) cohort (Table 2), which was initially used for linkage analysis of cortical cataract [13]. To select individuals for re-sequencing, we identified families linked to markers on 1p36 from our previous set. We re-sequenced all 17 exons and intron-exon boundaries of *EPHA2* in 34 individuals from the linked families (Table S1). We identified four new coding variants (*Ser74Ser*, *Arg721Gln*, *Lys728Lys*, and *Ile779Ile*) and one new non-coding variant (*A/G* in intron 12-13) (Table 3). These variants were rare (minor allele frequency MAF<1%) and have never been reported before. We also confirmed one non-coding insertion-deletion polymorphism (rs35519704: *-/G*) and seven other coding variants, including rs6678618 (*Ala190Ala*), rs6678616 (*Leu191Leu*), rs35484156 (*Ser277Leu*), rs2230597 (*Pro329Pro*), rs55655135 (*Leu632Leu*), rs10907223 (*Leu661Leu*), and rs3754334 (*Ile958Ile*). These polymorphisms were previously reported in public databases.

**Table 2:** Phenotypic characteristics of the BDES, BMES, and UKTS samples used for genotyping.

Study and Covariate	Cortical	Severe Cortical^a^	
		UA	AFF
BDES			
Total, N	1401	495	142
Average age at baseline (years)	64 (11.0)	61 (10.8)	70 (8.3)
Gender (% of female)	54.7	48.1	69.7
% of ever smokers	54.0	59.0	52.1
% of history of heavy drinkers	25.7	26.8	16.4
% of history of diabetes	16.0	12.0	19.0
Average body mass index (kg/cm2)	30.3 (5.8)	30.2 (5.9)	29.7 (5.7)
Average of age-related macular degeneration score	4.8 (3.2)	4.1 (2.7)	5.6 (3.5)
Average cortical cataract score (0–100%)	7.6 (11.9)	0.5 (0.3)	38.1 (12.1)
UKTS			
Total, N	185	103	53
Average age at baseline (years)	65 (4.5)	64 (3.7)	66 (4.9)
Gender (% of female)	100.0	100.0	100.0
% of ever smokers	44.3	46.6	39.6
Average alcohol intake	1.9 (1.1)	1.8 )1.2)	2.0 (1.0)
Average systolic blood pressure	132 (19)	129 (17)	137 (22)
Average cortical cataract score (0–100%)	16 (23.2)	0.0 (0.0)	47.8 (18.5)
BMES			
Total, N	1470	400	52
Average age (years)	64 (8.2)	70 (8.4)	72 (7.2)
Gender (% of female)	43.3	49.8	26.9
% of ever smokers	49.3	50.3	36.5
% of hypertension	54.1	59.2	63.5
Average systolic blood pressure	149 (21)	151 (21)	154 (20)
Average of age-related macular degeneration score	8.2 (2.7)	7.5 (2.9)	7.0 (3.0)
Average cortical cataract score (0–100%)	3.2 (7.6)	0.1 (0.1)	34.7 (9.4)

Values are given as mean (standard deviation [SD]) when appropriate.

a Severe cortical cataract represents a binary trait with a cutoff cortical score 25% for affected and with cortical scores<1% for unaffected in BDES, BMES, and UKTS. Severe cortical cataract is a subset of the cortical cataract phenotype. UA: unaffected; AFF: affected.

**Table 3:** Variants identified through re-sequencing.

Region	RS# or Amino Acid Change	Position^a^	SNP Type	Alleles	Amino Acid Change
Exon 3	Ser74Ser	16,348,061	Synonymous	T/A	S74S
	rs6678618	16,347,713	Synonymous	G/A	A190A
	rs6678616	16,347,710	Synonymous	G/A	L191L
Intron 3-4	rs35519704	16,347,431	Boundary of Exon 3	-/G	-
Exon 4	rs35484156	16,337,506	Non-synonymous	C/T	S277L
Exon 5	rs2230597	16,337,260	Synonymous	C/T	P329P
Exon 11	rs55655135	16,332,419	Synonymous	G/A	L632L
	rs10907223	16,332,332	Synonymous	C/T	L661L
Intron 12-13	-	16,331,400	Boundary of Exon 12	A/G	-
Exon 13	Arg721Gln	16,331,308	Non-synonymous	G/A	R721Q
	Lys728Lys	16,331,137	Synonymous	G/A	K728K
Exon 14	Ile779Ile	16,330,598	Synonymous	C/T	I779I
Exon 17	rs3754334	16,324,354	Synonymous	G/A	I958I

a Positions are based on NCBI Build 36.3.

Among variants identified via re-sequencing, two non-synonymous variants (*Arg721Gln* and rs35484156) and two synonymous variants (*Ile779Ile* and rs6678616) were pursued by genotyping all available individuals (Table 4) in the discovery BDES set (494 families, N = 1401). The *Arg721Gln* variant demonstrated the strongest association (P values: cortical = 2×10^−8^, severe cortical = 8×10^−5^) in the BDES. We performed extensive quality control metrics for the final model containing *Arg721Gln* in the discovery dataset to confirm both the consistency and the validity of the association result (Figure S4). We did not discover any bias in the SNP association caused by other factors.

**Table 4:** Characteristics of SNPs in the *EPHA2* gene.

SNP	Position	Context	Alleles	MA	RA	MAF	Platform	Selection Criterion
rs924201	16,318,936	Downstream	G/A	A	A	0.383	SNPlex	Non-coding conserved
rs7548209	16,321,209	Downstream	G/C	G	C	0.333	SNPlex	Non-coding conserved
rs1803527	16,324,000	3′UTR	T/C	C	C	0.017	SNPlex	Non-coding conserved
rs3754334	16,324,354	Exon17-Syn	G/A	A	A	0.292	TaqMan	Tag-based
rs11260721	16,327,639	Intron 16-17	G/A	G	G	0.188	SNPlex	Non-coding conserved
Ile779Ile	16,330,598	Exon 14	C/T	T	T	0.019*	TaqMan	Re-sequencing
Arg721Gln	16,331,308	Exon 13	G/A	A	A	0.003*	TaqMan	Re-sequencing
rs13375644	16,334,420	Intron 6-7	G/A	A	A	0.092	SNPlex	Non-coding conserved
rs2230597	16,337,260	Exon5-Syn	G/A	A	A	0.412	TaqMan	Tag-based
Ser277Leu	16,337,506	Exon 4	C/T	T	T	0.005*	TaqMan	Re-sequencing
rs11260745	16,338,590	Intron3-4	A/G	G	G	0.085	SNPlex	Non-coding conserved
rs3768293	16,340,511	Intron3-4	G/T	G	G	0.367	TaqMan	Tag-based
rs6603867	16,347,288	Intron3-4	T/C	T	C	0.375	TaqMan	Non-coding conserved
rs6678616	16,347,710	Exon3	G/A	A	A	0.333	SNPlex	Re-sequencing
rs1472408	16,351,241	Intron2-3	C/T	C	T	0.367	SNPlex	Non-coding conserved
rs6603883	16,355,563	Promoter	C/T	T	T	0.358	SNPlex	Non-coding conserved
rs11260822	16,358,398	Upstream	G/A	A	A	0.356	SNPlex	Non-coding conserved
rs904106	16,359,380	Upstream	C/T	T	T	0.017	TaqMan	Tag-based
rs729402	16,361,693	Upstream	A/G	A	G	0.393	SNPlex	Non-coding conserved

Positions are based on NCBI Build 36.3; alleles shown are on the coding strand (- strand); MA: minor alleles; RA: reference alleles that were used in genetic models for association tests; MAF: minor allele frequency in dbSNP database; cells with asterisks (*) represent simple average MAF estimated from the three samples, the BDES, BMES, and UKTS.

The risk allele for *Arg721Gln* was the rare ‘*A*’ allele (Table 5). We estimated that after adjusting for covariates the risk allele for *Arg721Gln* can cause a net increase in the cortical score of 9.2% under the dominant model (i.e. if an individual carries one ‘*A*’ allele for the *Arg721Gln* variant, the cortical cataract score increases by 9.2% compared to carrying ‘*GG*’ alleles). Since we did not have *Arg721Gln* ‘*AA*’ homozygotes in our data, we did not assess the effect size for a recessive model. Using the severe cortical cataract trait the association signal at *Arg721Gln* was less significant (Table 5), showing that smaller sample sizes led to reduced power.

**Table 5:** P values from rank transformed traits and effect sizes from the quantitative cortical scores (β) of the risk allele at markers under the dominant model in *EPHA2* for each separate study and for the joint analysis of all studies.

Trait and SNP	RA^a^	Individual Study Analysis (β)^b^				Meta-Analysis (Z-score)^c^	
		BDES	UKTS	BMES	BMES Trend	Family Set	All
Cortical Cataract Score							
rs7548209	C	0.4516	0.0524	0.0365 (0.85)	-	0.1561	0.0747
rs3754334	A	0.3935	0.0252 (2.95)	0.0734	-	0.1205	0.0180 (2.37)
*Arg721Gln*	A	2×10^−8^ (9.20)	MS	NA	-	-	-
rs3768293	G	0.0016 (1.24)	0.0439 (2.94)	0.2705	-	3×10^−4^ (3.65)	9×10^−4^ (3.34)
rs6603867	C	1×10^−4^ (1.58)	0.8325	0.2411	-	4×10^−5^ (4.13)	0.7110
rs6678616	A	7×10^−5^ (1.24)	0.5249	0.1520	-	1×10^−4^ (3.90)	1×10^−4^ (3.87)
Haplotype							
rs7548209-rs3754334	CG	-	-	0.0024 (2.36)	-	-	-
rs7548209-rs3754334	GG	-	-	0.0202 (−0.74)	-	-	-
rs6603867-rs6678616	CA	-	-	1×10^−6^ (6.57)	-	-	-
Severe Cortical Cataract							
rs7548209	C	4×10^−6^	3×10^−4^	0.1051	0.0027	8×10^−9^ (5.78)^d^	0.3884
rs3754334	A	0.0225	1×10^−4^	0.0196	0.0065	2×10^−4^ (3.70)^d^	0.1602
*Arg721Gln*	A	8×10^−5^	MS	NA	-	-	-
rs3768293	G	7×10^−6^	0.0040	0.0433	0.0140	9×10^−8^ (5.34)^d^	0.0037 (2.91)^d^
rs6603867	C	2×10^−5^	0.0150	0.0435	0.0265	7×10^−5^ (3.97)	0.0409 (2.04)
rs6678616	A	0.0215	0.0152	0.3788	0.0452	0.0014 (3.19)^d^	0.0271 (2.21)

Cells with a dash indicate that the analysis was not performed.

a RA represents risk alleles (reference alleles).

b MS and NA represent monomorphic SNPs and SNPs that were not analyzed because of small variance under the dominant model, respectively. The Cochran-Armitage (additive) trend test (BMES Trend) was also used to conduct a case-control association test of individuals age-matched for severe or no cortical cataract in the BMES. Parentheses in each individual study for cortical cataract represent effect sizes (β).

c Parentheses in meta-analysis represent Z-scores.

d In the meta-analyses, these SNPs for severe cortical cataract show significant P values for heterogeneity tests of effect sizes (Q test P<0.05).

**Figure 3:**
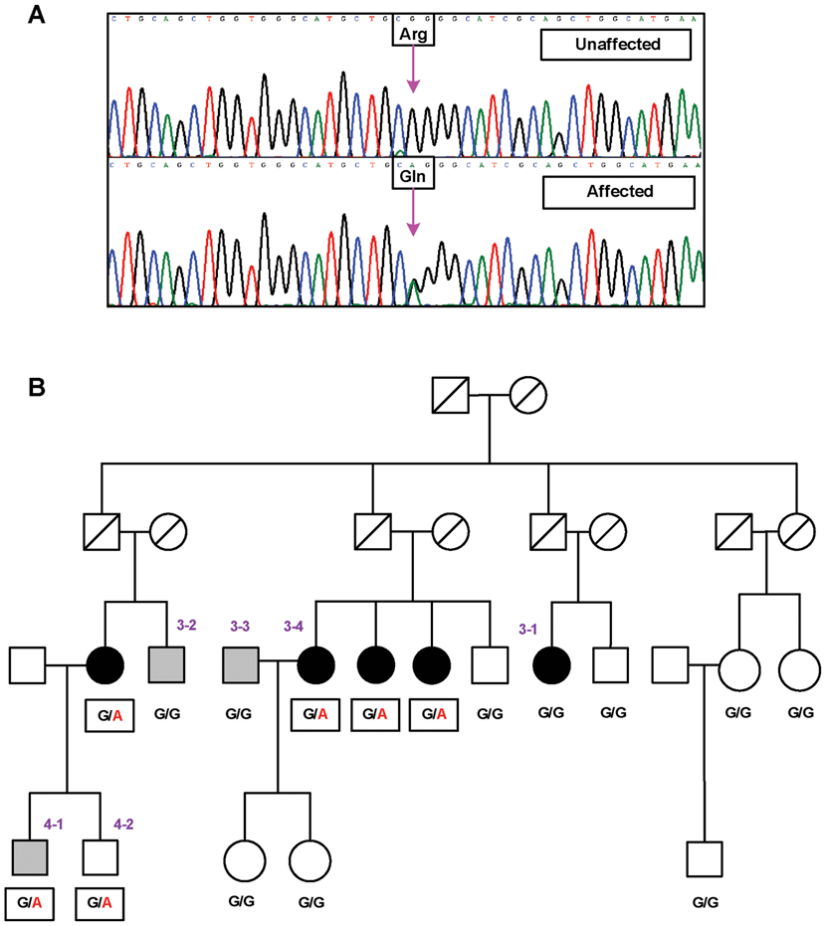
Characterization of a new coding variant *Arg721Gln* in exon 13. (A) An example electropherogram of the coding strand is displayed for an affected and an unaffected individual, with the arrow marking the variant. Individuals with disease were heterozygotes (CAG) for Gln/Arg while unaffected individuals were homozygotes for Arg/Arg (CGG). (B) A large family with cortical cataract was examined and the co-segregation of *Arg721Gln* with cortical cataract determined. Two members (3-1 and 3-2) and one marry-in (3-3) were GG (Arg/Arg) but have cortical cataract, suggesting phenocopies. Symbols filled with black or gray represent those affected with severe (≥25% of the lens affected) or moderate (5%≤score<25%) cortical cataract, respectively. In the parent generation, all heterozygotes for the *Arg721Gln* variant developed severe cortical cataract or have had cataract surgery. However, this variant has age dependent penetrance, as individuals who developed severe disease are older (e.g. individual 3-4 who is 80 years old). Individuals in the offspring generation who are at risk may not have developed full-fledged cortical cataract (e.g. 4-1 who is less than age 65 with moderate cataract, or 4-2 who is a carrier but remains free of disease at age 63).

The sequencing data for *Arg721Gln* confirmed that the risk allele ‘*A*’ segregates with disease in a BDES family (Figure 3). We were unable to investigate segregation for heterozygous individuals in the other families, because missing data caused these individuals to be singletons or unconnected. We also genotyped the *Arg721Gln* variant in two replication datasets (Table 2), the United Kingdom Twin Eye Study (UKTS; 185 individuals from 172 families) and the Australian Blue Mountains Eye Study comprising unrelated persons (BMES; N = 1470). The three datasets were comparable with respect to measurement of the trait and the way the data were defined for analysis (Figure S5). The frequency of the rare ‘*A*’ allele is 0.6%, 0% and 0.2% in the BDES, UKTS, and BMES, respectively. These frequencies are not significantly different in the BMES and UKTS (Table S2); increased frequency of the ‘*A*’ allele in the BDES was probably due to enrichment of the same risk allele in these families (i.e. a founder effect), and we believe that 0.1%–0.2% represents a true population estimate for this rare variant. The risk allele has 78% penetrance in heterozygous individuals whose age is 70 years or more. We were unable to test association for the *Arg721Gln* in the replication sets because either the rare allele was not present (UKTS) or its frequency was too small (BMES) to be used in the statistical models.

### Biochemical and Cellular Characterization of the Non-Synonymous Variant *Arg721Gln*

**Figure 4:**
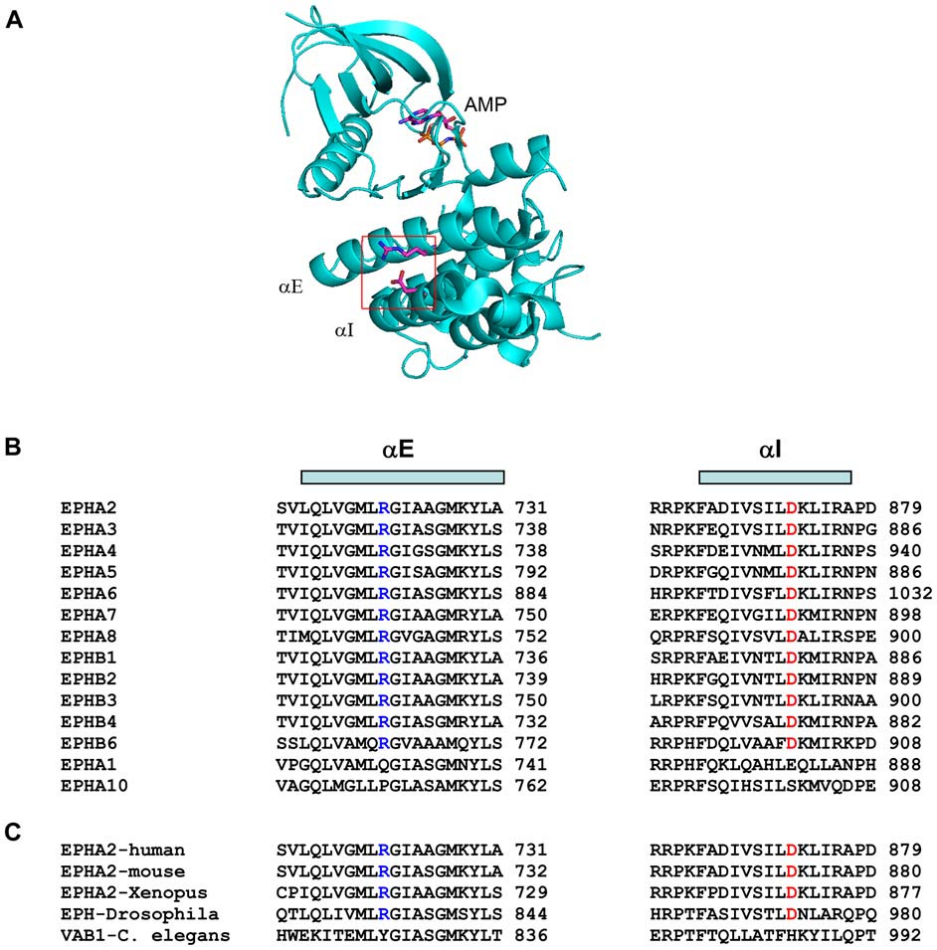
Concordant conservation of Arg721 and Asp872 in EPH kinase domains. (A) Examination of crystal structure of EPHA2 kinase domain reveals that Arg721 in αE forms a salt bridge with Asp872 in αI. (B) Concordant conservation of Arg721 and Asp872 in different members of human Eph kinases. Note that *EPHA9* and *EPHB5* are not present in human genome and are not shown. (C) The same residues are also concordantly conserved across different species.

**Figure 5:**
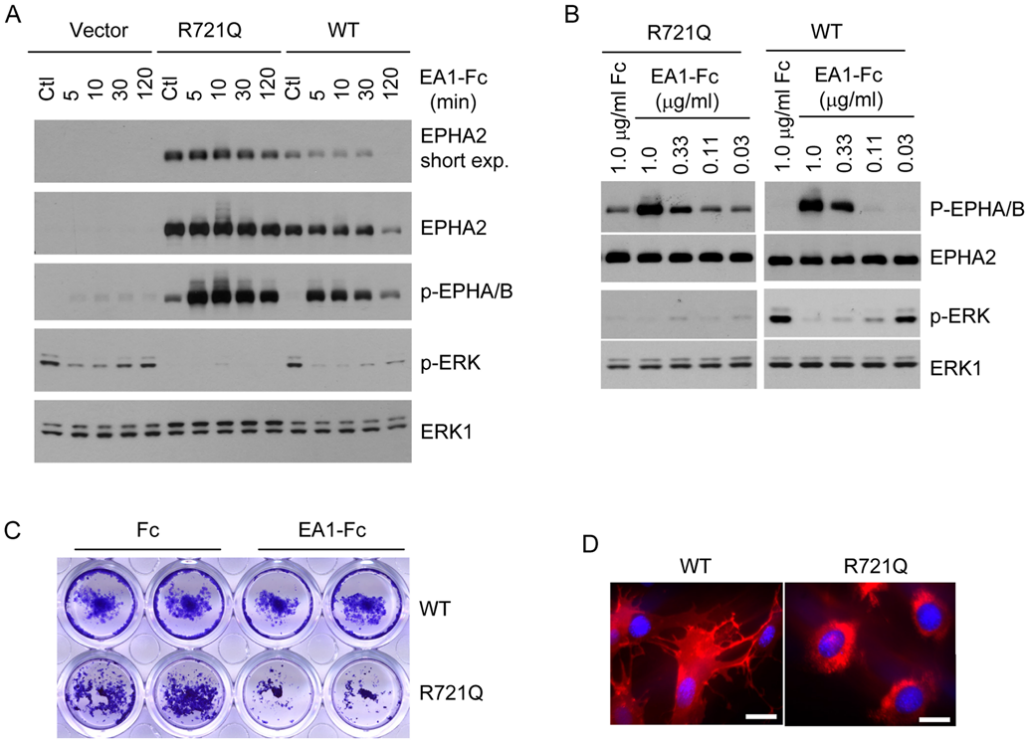
Functional characterization of *Arg721Gln* variant associated with human cataract. (A,B) *Arg721Gln* mutation causes an increased basal activation of EPHA2 kinase, which was correlated with dramatically reduced basal ERK1/2 activities. In a kinetic study (A), HEK 293 cells expressing WT-, *Arg721Gln*-EPHA2 or vector control were stimulated with 2 µg/ml ephrin-A1-Fc for the indicated times. In a separate experiment, a dose-response study was carried out (B), where different doses of ephrin-A1-Fc were used to stimulate cells expressing WT- or *Arg721Gln*-EPHA2 for 10 min. Cell lysates from both experiments were blotted with the indicated antibodies as described previously [53]. (C) HEK 293 cells expressing Arg721-Gln mutant EPHA2 but not WT-EPHA2 were growth-inhibited by ephrin-A1 in a clonal growth assay as described previously [26]. About 200 cells/well were seeded in a 24-well culture dish and cultured for 10 days in the presence or absence of ephrin-A1. (D) Stochastic intracellular trapping of *Arg721Gln* mutant in MEF cells derived from *Epha2* knockout embryos. Shown is a cluster of cells with the mutant EPHA2 trapped inside the cells. In contract, WT-EPHA2 was primarily expressed on the cytoplasmic membrane. Scale bar: 5 µm.

Functionally, the *Arg721Gln* mutation changes arginine, a positively charged amino acid, to glutamine. Examination of the EPHA2 kinase domain crystal structure [25] revealed that Arg721 in the αE helix forms a salt bridge with Asp872 in the αI helix (Figure 4A). Interestingly, both Arg721 and Asp872 are concordantly conserved among different members of human Eph kinases and among EPHA2 proteins across different species (Figure 4B and 4C). The *Arg721Gln* mutation may disrupt the salt bridge and affect its conformation and function. To test this possibility, wild type (WT) and *Arg721Gln* mutant EPHA2 were expressed in HEK 293 cells that have low endogenous EPHA2 [26]. Although the mutant receptor still remained responsive to ligand stimulation in both kinetic and dose-response studies (Figure 5A and 5B), it displayed significantly higher basal activation than WT-EPHA2 in the absence of ligand stimulation. Consistent with our previous report that activated EPHA2 inhibited the Ras/ERK1/2 signaling cascade [26], the higher basal activation of EPHA2 was correlated with dramatically reduce basal ERK1/2 activities compared to WT-EPHA2 or vector control, suggesting altered signaling by the mutant EPHA2. In a clonal growth assay, HEK 293 cells expressing *Arg721Gln-*EPHA2 were significantly growth-inhibited cells by ephrin-A1, whereas WT-EPHA2 expressing cells were refractory (Figure 5C). In addition, we observed stochastic intracellular retention of the *Arg721Gln* mutant EPHA2, but not WT-EPHA2 when expressed in mouse embryonic fibroblast (MEF) cells derived from *Epha2^−/−^* embryos, affecting about 40% of total cell populations (Figure 5D). The effects were cell type-specific, as they were not observed in HEK 293 cells. While the molecular mechanism for the cytosolic retention is unclear at present, the data in aggregate demonstrate that the *Arg721Gln* mutation significantly alters EPHA2 signaling and cellular regulation in vitro. Because EPHA2 is essential in maintaining lens clarity in the mouse knockout model (Figure 1), it is possible that such perturbation of EPHA2 functions by the *Arg721Gln* mutation can predispose the carrier to cataractogenesis. Further studies, including knock-in of the mutant allele in mice, will be necessary to determine if the mutation is sufficient to predispose to lens opacity in vivo.

### Identification of Common Variants Associated with Age-Related Cortical Cataract

**Figure 6:**
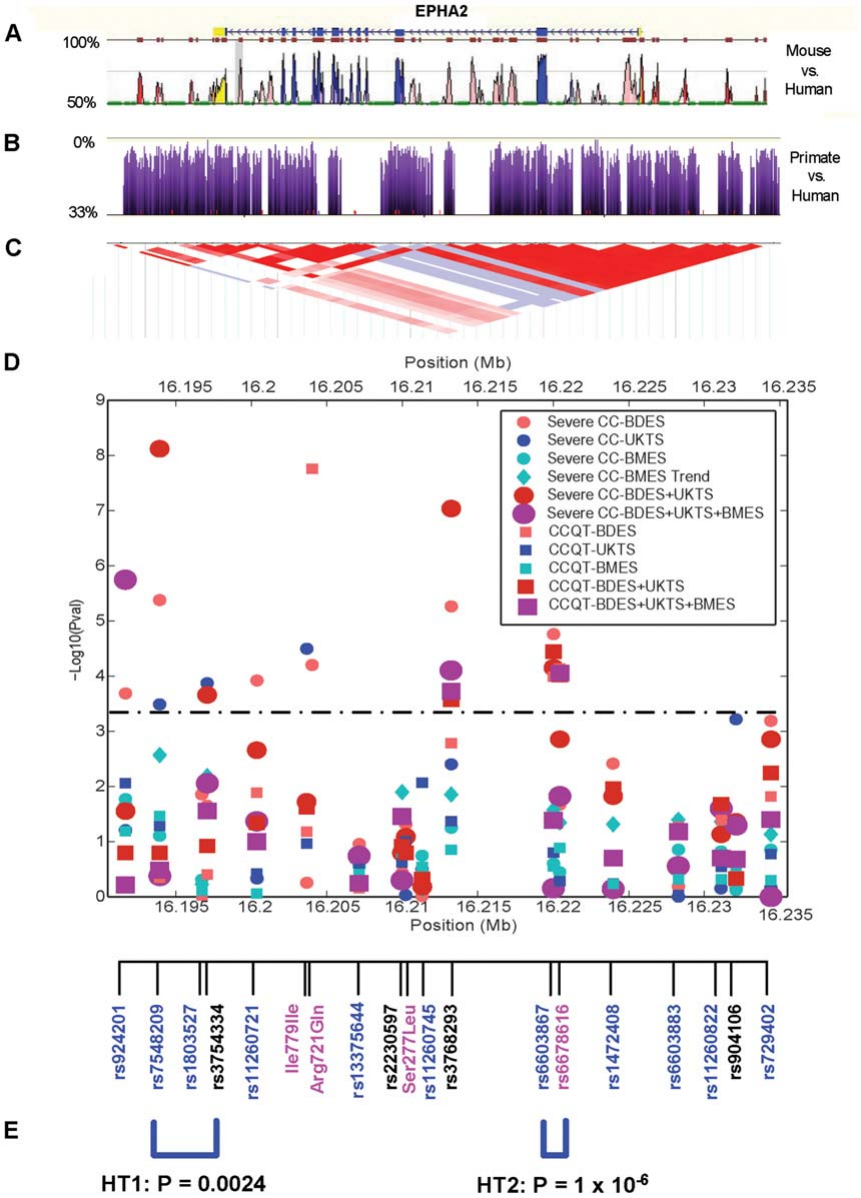
Single SNP and haplotype association results for *EPHA2*. Markers were selected based on phylogenetic footprinting in mouse-human (A), or phylogenetic shadowing in human-primates (B), or a tag-based approach using LD from the CEU HapMap sample (C). Markers in blue are in phylogenetically conserved areas, in black are tag-based, and in pink are identified via re-sequencing (D). Single marker results are reported using the full range of quantitative cortical cataract scores (CCQT) and the extreme phenotypic contrast for cortical cataract (severe CC), from the BDES, BMES, and UKTS. Meta-analysis results are shown using the two family studies (BDES and UKTS) and using all three studies (BDES, BMES, and UKTS). We plotted −log_10_(P value) from the likelihood ratio test for CCQT and severe CC at each marker (D). The dashed line represents the 0.05 P value after adjusting for multiple testing for the genotyped SNPs and multiple models (0.05/[19 markers×3 genetic models×2 traits] = 4×10^−4^). (E) Window sizes of 2–5 markers were used to identify the most significant haplotypes in the 3′ (HT1) and the 5′ (HT2) region using percent of the lens affected by cortical cataract in the BMES, the sample of unrelated persons.

In order to find common variants associated with disease, we selected 15 SNPs based on tagging parameters and phylogenetic conservation (Figure 6A–6C) in addition to 4 coding variants identified via re-sequencing, resulting in 19 additional SNPs that covered at least 90% of the gene (Table 4). We genotyped these SNPs in three worldwide Caucasian populations (Table S2). Genotyping in all three datasets demonstrated that the *EPHA2* gene contained at least two blocks of linkage disequilibrium (LD), one at the 5′ and another at the 3′ end that were relatively independent; the LD in the BDES, BMES, and UKTS was very similar to that in the CEU HapMap sample (Figure S6). The markers within each block are highly correlated, while those between the 5′ and 3′ blocks show low correlation (Figure S6). The highest pairwise correlation (r^2^) between SNPs in 5′ and 3′ LD blocks did not exceed 0.5 in all three datasets (Figure S6).

We performed association analyses using percent of lens affected with cortical cataract as a dependent variable. We also tested association using affection status only, classifying individuals as clearly affected (percent of cortical score ≥25) or unaffected (percent of cortical score <1), and eliminating all those with intermediate scores. If the whole range of scores was considered, the average cortical score in the BDES, UKTS, and BMES was 7.6%, 16.3%, and 3.2%, respectively, while the proportions with severe cortical cataract in the BDES, UKTS, and BMES were 10%, 29%, and 3%, respectively. Prior to association analysis, the quantitative trait of percent of lens affected and the binary trait, affected with cataract (yes/no), were adjusted for significant covariates using regression. The values obtained after regression (residuals) were inverse rank transformed to normalize their distribution. Association testing was performed on values of the trait before and after inverse rank transformation. Multiple genetic models (additive, dominant, and recessive) were tested for each SNP (Table S3 and Table S4), but for brevity only the dominant models are presented in the main text (Figure 6 and Table 5); the remainder of the models are presented in the supplementary materials. In the discovery family data, six SNPs, rs924201, rs7548209, rs11260721, rs3768293, rs6603867 and rs6678616 showed significant association (nominal P<4.4×10^−4^, calculated as 0.05 divided by 3 models×2 traits×19 genotyped SNPs), either with the full range of cortical cataract scores or with severe cortical cataract. Except for rs6678616, P values at the other five SNPs (rs924201, rs7548209, rs11260721, rs3768293 and rs6603867) were smaller when using the categorical yes/no definition of cortical cataract, despite the decrease in sample size (Table 5). Differences in the ability to detect association as a result of changed in phenotypic definition are not unexpected, as association is dependent on allele frequency, as well as on sample size.

Because the average cortical cataract score of the cases in the UKTS was 10% greater than the other two studies, the proportion of severe cases in the UKTS is 3 times and 10 times higher than that in the BDES and BMES, respectively (Table 2). Thus, in the replication dataset from the UKTS, P values for the severe cortical cataract trait were in general smaller than the full range of cortical cataract scores (Figure 6). Four of the same common SNPs observed in the BDES, rs7548209, rs3754334, rs3768293, and rs6678616, showed replication (smallest P value with rs3754334 = 1×10^−4^). In the UKTS we also identified significant association for severe cortical cataract with another rare variant Ile779Ile (P = 3×10^−5^). Rigorous diagnostic testing of the final models in the two family datasets (BDES and UKTS) confirmed that the results were valid (Figure S4).

### Association Analysis of Common Variants in Unrelated Data

We conducted single SNP and haplotype association analyses in the BMES (Table S5). As the BMES cohort is slightly younger, if we selected an age-matched case-control set, restricting controls to 70 years or greater, and defining cases as those with a cortical score≥25% and controls with cortical score<1% (Table 5) we observed replication using the trend test (best P value at rs7548209 = 0.003). Without these restrictions, the association was less significant because the chance of misclassifying disease is higher at younger ages (Table 5). Using the five most significant SNPs, rs7548209, rs3754334, rs3768293, rs6603867 and rs6678616, we performed a haplotype association test for cortical cataract using a moving window approach with different window sizes from 2 to 5. Haplotype analyses showed stronger evidence than single marker tests in the BMES (Table 5). Three haplotypes showed significant association at a nominal P<0.05 with window size 2 (Table 5). The most significant haplotype encompassed rs6603867-rs6678616 (r^2^ = 0.7) with *C-A* as the risk haplotype (β = 6.6, P = 1×10^−6^) and explained 6% of the total variance in cortical cataract scores. This result was consistent with the single SNP association tests in the two family studies (Figure 6E). The next best association is with *C-G* as the risk haplotype (β = 2.4, P = 0.0024) and *G-G* as the protective haplotype (β = −0.7, P = 0.020) at markers rs7548209-rs3754334 (r^2^ = 0.8), suggesting that the ‘*C*’ allele at rs7548209 causes increased risk; this haplotype explained 2% of the variance in the quantitative trait for cortical cataract. In total, these haplotypes accounted for 8% of the total variation. The risk alleles in the haplotype association test above are similar to those in the single SNP association in the two family datasets (Table 5).

### Heterogeneity Tests and Meta-Analysis

Joint (meta-)analysis was conducted to combine the results of the two family studies and then to combine all three studies (Table S6). The most significant association for the full range of cortical cataract trait values using the two family sets and all three datasets was found with rs6603867 (P = 4×10^−5^) and rs6678616 (P = 1×10^−4^), respectively (Table 5). There was no significant heterogeneity for cortical cataract scores under the dominant model (P>0.05) with these two significant SNPs (Table S6A). For severe cortical cataract, rs7548209 (P = 8×10^−9^) and rs3768293 (P = 0.0037) were the most significant markers when combining the two family datasets and when combining all three datasets, respectively (Table 5). We noted that the SNPs for the severe cortical cataract trait showed heterogeneous effect sizes across studies (Table 5). Nevertheless, the direction of the association for the full spectrum of cortical cataract was the same among all three studies (Tables S3, Table S4, and Table S5). Diversity in effect sizes among the three datasets for severe cortical cataract is not surprising, because the proportion of severe cases in each study is different (Table 2). As a result, meta-analysis of all three datasets did not improve the overall significance of the results (Table 5). However, single SNP association, haplotype association, and meta-analysis consistently identified that two independent association signals are present in the 3′ (rs7548209 and rs3754334) and the 5′ (rs3768293, rs6603867, and rs6678616) regions of the gene. The maximum pairwise correlations between SNPs in the 3′ and the 5′ regions is less than 0.4 (Table 5), arguing for more than one susceptibility variant being present in these samples.

## Discussion

We report here that *EPHA2* is essential in maintaining the clarity of crystalline lens in aging mice and is associated with age-related cortical cataract in humans. EPHA2 protein is expressed in the cortical lens fiber cells, and homozygous deletion of *Epha2* in mice caused progressive cortical cataract. At the molecular level, cataractogenesis was preceded by accumulation of underphosphorylated HSP25, indicating elevated stresses and protein misfolding in *Epha2* ^−/−^ lens. In human studies involving three independent Caucasian populations, we discovered common polymorphisms, as well as a rare variant that significantly altered the function of EPHA2 kinase activities and cellular functions. Therefore, converging evidence from genetically engineered mice and human population studies, coupled with in vitro cell-based assays, strongly suggest that *EPHA2* is the first major genes associated with the common form of cataract. The involvement of a receptor tyrosine kinase in cortical cataract etiology points to future directions in designing new preventive and therapeutic approaches for cortical cataract by targeting *EPHA2*.

Prior to this study, the only noticeable phenotype reported in *Epha2* knockout mice is the kinked tails, which develops during early embryogenesis [20]. For yet unknown reasons, the penetrance of this phenotype has been quite low in the large cohorts of the same strain of knockout mice maintained in our facility on either FVB/NJ or C57Bl/6 genetic background. In another stain of *Epha2* knockout mice generated by the secretory trapping strategy [17],[18], the incidence of kinked tails was also very low. This is in contrast with cataract phenotype reported here that is observed in significant fraction of both strains of homozygous knockout mice.

There are several unique approaches in our study compared with other studies to identify genes involved in complex disease. First, we utilized two independent lines of *Epha2* knockout mice that were extensively investigated for potential molecular implications in cataractogenesis. This effort provided confidence in subsequent studies pursuing causal variants in human populations. Second, we investigated association with polymorphisms of *EPHA2* in worldwide Caucasian populations from the United States, United Kingdom, and Australia. Phenotypic measurements were comparable among the three datasets, but there was variability in the extent of severity of cataracts in these populations. The most severe forms of cataract were associated with rare variants that were unique to each population and there was little replication between populations. Common variants could be replicated across populations with a greater degree of certainty. To ensure validity of the results, we extensively examined diagnostics of the final models in the family data. A candidate gene study using family data, unlike a genome-wide association study using unrelated data, requires special techniques to ensure the validity, i.e. normality of the model residuals expected asymptotically and required for the P values that presume normality to be valid. We applied the George-Elston transformation to both sides of the regression equation in the association tests [27]. We confirmed the normality of the residuals in the final models for all SNPs (Figure S4).

Our studies led to the successful identification of a non-synonymous variant, *Arg721Gln*, residing in the protein kinase domain of EPHA2. Moreover, the variant showed increased spontaneous activation in the absence of ligand stimulation, suggesting that the coding variant alters the spontaneous and ligand-triggered cellular signaling. The altered signaling is associated with enhanced growth inhibition by ligand, and stochastic intracellular retention in primary fibroblasts. The reason for the cytoplasmic retention in only some, but not all cells expressing the mutant is not clear at present. Regardless of the mechanisms, cytoplasmic trapping is likely to interrupt both signaling and structural functions of EPHA2/ephrinA system in the lens fiber cells. Because EphA2 is essential for maintaining lens clarity as evidenced by cataractogenesis in homozygous knockout mice, functional alterations in EphA2 is likely to impair its physiological roles in the lens, predisposing the carriers to increased risk of cataract development.

In addition to this rare variant, common polymorphisms were also discovered and replicated in worldwide Caucasian populations, suggesting that *EPHA2* could be involved in predisposition to cataractogenesis in the general population. The most significant common variant for the quantitative cortical cataract trait was rs6678616 in the region coding for the ligand binding domain of the *EPHA2* gene. Bioinformatic investigation revealed that rs6678616 is located in an exonic splicing enhancer element, and nucleotide changes of the SNP may potentially affect the affinity of splicing enhancer factors up to 14-fold (data not shown). The most significant SNP, rs7548209, for severe cortical cataract is present in a downstream conserved non-coding region of the *EPHA2* gene. Characteristics of this variant are not known. It is possible that such a variant may affect *EPHA2* expression during aging.

While this manuscript was under preparation, it was reported that mice with homozygous deletion of *Efna5* encoding a ligand (ephrinA5) for EPHA2 also developed cataract [28]. These data nicely complement the data described in this paper, and demonstrate that both the receptor (EPHA2) and the ligand (ephrinA5) are required for maintaining lens clarity. In addition, recent genetic studies involving a relatively small number of affected individuals have found a link between congenital cataract and variants of *EPHA2*, although none of rare variants were functionally characterized [29]. These recent results on rare congenital cataract support our conclusion on the link between *EPHA2* and the much more common age-related cataract, based on large human population studies and genetically engineered mice.

Since aging was the most important risk factor for age-related cataract, age-dependent reduction of EPHA2 protein expression in normal lens and age-dependent deterioration of lens clarity in *Epha2* knockout mice suggests an important role of EPHA2 kinase in cataractogenesis during aging. Previous studies have shown that EPHA2 regulates epithelial cell morphogenesis and homeostasis in part by interacting with components of adherens and tight junctions, such as claudins and cadherins [30]. Notably, the lens is primarily composed of lens fiber cells where intricate cell-cell interactions are essential for structural and functional integrity. Changes of EPHA2 expression or function due to aging or genetic predisposition can impair junctional structure, leading to excessive cellular stress and eventual opacity.

Recent studies show that there are common genes and pathways among late onset neurodegenerative diseases, including Alzheimer's disease, multiple sclerosis, diabetes, and age-related eye disease. Genes involved in neurodegenerative diseases, kinesin light chain 1 [11] and apolipoprotein E [12], showed association with age-related cataract. Also, there is evidence of a link between longevity and age-related cataract [31]. Successful aging, defined as preserved cognition, is associated with reduced age-related cataract among the elderly [31]. Microarray expression analysis proved that lens expresses brain specific molecules, including β-amyloid secretases and degrading enzyme [32], synapsin and synaptic vesicle protein [33], and brain-specific miRNAs [34]. This suggests that common genes and pathways may be involved in both age-related cataract and neurodegenerative diseases affecting the elderly population. Since the EPH/ephrin system is known to be critical for neural development [35] and vision processes in the midbrain [36], the *EPH* and *EFN* genes may be potentially involved in a broad spectrum of age-related eye and neurodegenerative diseases. Further functional investigation of these genes will elucidate pathogenic mechanisms leading to age-related cataract and other late onset diseases.

In summary, converging evidence from mouse and humans demonstrates that *EPHA2* has an important role in maintaining lens transparency. While genetic predisposition is known to be a major contributing factor to age-related cortical cataract, our study has identified and characterized genetic variants of the *EPHA2* gene in human, and both common and rare variants confer risks for cortical cataract.

## Materials and Methods

### Animals

The KST085 line of *Epha2* knockout mice was generated through secretory gene trapping as described previously [18]. The secretory trapping vector was inserted at the boundary between exon 5 and intron 6, leading to the truncation of exons 6 to 17 encoding the second fibronectin type III repeat in the extracellular domain all the way to the carboxyl terminal end. The remaining ectodomain encoded by exons 1 to 5 is fused to neomycin resistance and β-galactosidase reporter cassette (β-geo) and is trapped inside the cells in secretory vesicles, presumably in inactive form. These mice, which were generated on a C57Bl/6/129 genetic background, were then backcrossed with FVB/N mice. Mice from N4 or higher backcrosses were bred with each other to generate *Epha2^+/+^*, *Epha2^+/−^*, and *Epha2^−/−^* mice that were used in subsequent studies. Another line of mice on C57Bl/6 background was provided by Drs. M. Asano and Y. Iwakura [20]. In these mice, a retrovirus was inserted in the first codon of *Epha2*, leading to abolishment of EPHA2 expression.

All procedures involving mice were performed in accordance with guidelines set forth by the American Association for Accreditation of Laboratory Animal Care and the USPHS “Policy on Humane Care and Use of Laboratory Animals.” Studies were approved and supervised by The Case Western Reserve University Institutional Animal Care and Use Committee.

### Histological Analysis and Immunofluoresence

For histological analyses, mouse eyes were enucleated and fixed in neutral buffered 10% formalin solution for 24 hours. Eyes were dehydrated through a graded alcohol series and embedded in paraffin. Sections (5 µm) were cut through papillary-optic nerve axis and stained with hematoxylin and eosin (H&E).

For immunofluorescence analyses, postnatal day 14 eyes were dissected, fixed with 4% paraformaldehyde for 25 min. Eyes were washed with ice cold Phosphate Buffered Saline (PBS) and kept in 15% sucrose (prepared in PBS) overnight. Tissue freezing medium (Triangle Biomedical Science, Durham, NC) embedded eyes were cut on a cryostat (Leica, Germany), and air dried onto Superfrost Plus slides (Fisher Scientific). After washing with PBS, the sections were blocked with 50 mmol/L NH_4_Cl and permeabilized with 0.3% NP40 for 10 minutes. The sections were then incubated with primary antibodies at room temperature for 1 hour followed by detection with donkey secondary antibodies conjugated with FITC or Texas-red (Jackson ImmunoResearch, West Grove, PA) at room temperature for 30 minutes.

EPHA2 and Ephrin-A1 proteins were detected in human and mouse lens paraffin-embedded sections. Briefly, sections were deparaffinized, hydrated, and immersed in citrate buffer (10 mM sodium citrate, 0.05% Tween 20, pH 6.0) for 10 minutes at 95°C. Primary antibodies were incubated with samples and then detected by Biotinylated secondary antibody and avidin–biotin–peroxidase system (Vector). Color immunostaining was revealed using diaminobenzidine (Vector). Images were taken using a fluorescent Leica DM-IRE2 microscope equipped with a SPOT RT-SE digital camera (Diagnostic Instrument) and analyzed with MetaMorph software 6.1r4 (Universal Imaging).

Antibodies used include: goat anti-mouse EPHA2 ectodomain, goat anti-human EPHA2 (R&D Systems, Minneapolis, MN), rabbit anti-EPHA2 and anti-ephrin-A1, goat anti-HSP25 and mouse anti-phospho-ERK, rabbit anti-ERK (Santa Cruz Biotechnology, Santa Cruz, CA), rabbit anti-phospho-HSP25, anti-phospho-AKT, anti-Akt, anti-GAPDH (Cell Signaling), mouse monoclonal anti-N-cadherin (BD Biosciences). Rabbit-anti-α,β,γ-crystallin were kindly provided by Dr. Zigler (National Eye Institute).

### Tissue Extraction and Immunoblot Analysis

Lenses were dissected from eyes and homogenized in ice-cold lysis buffer containing 20 mmol/L Tris (pH 7.4), 125 mmol/L NaCl, 10% glycerol, 1% Triton X-100,0.5% DCA, 0.1% SDS, 20 mmol/L NaF, 1 mmol/L phenylmethylsulfonyl fluoride, µg/mL aprotinin, 4 µg/mL leupeptin, and 1 mmol/L Na_3_VO_4_. Then samples were centrifuged at 13,000 g for 10 minutes at 4°C. Protein concentrations in supernatant were assessed using the bicinchoninic acid protein assay kit (Bio-Rad, Hercules, CA). Equal amounts of protein extracts were resolved by 4–20% SDS-PAGE and electrotransferred onto polyvinylidene difluoride membranes (Millipore, Bedford, MA), which were then blotted with the indicated antibodies.

### Slit Lamp Biomicroscopy

Mice were anesthetized by an intramuscular injection of Tribromoethanol at 200 mg/kg body weight followed by induction of mydriasis with tropicamide (1%) and phenylephrine (10%). The lenses were examined with slit lamp and the observations were recorded by digital photography.

### Subjects

#### The Beaver Dam Eye Study

The Beaver Dam Eye Study (BDES) is a population-based cohort study for age-related ocular diseases and was used as a discovery sample set. Informed consent was obtained from the participants following a protocol approved by the Institutional Review Board (IRB) of the University of Wisconsin; the tenets of the Declaration of Helsinki were observed. The population, the methodology, and the baseline parameters are described in detail elsewhere [37]. Genome-wide linkage was previously examined in this cohort using microsatellite markers and we obtained evidence for a cortical cataract locus on 1p36 [13]. DNA and phenotypes were available for 1401 individuals in 494 families. Characteristics of genotyped individuals were not different from those in the rest of the cohort; therefore, the genotyped data can be extrapolated to the full cohort.

Retroillumination photographs were used to quantify cortical cataract. The lens photographs were graded based on the Wisconsin Cataract Grading System [37]. For grading cortical cataract, a grid was used with a central circle that occupies 2% of the entire lens surface, and subfields covered the remaining area, where each subfield was 12.25% of the total area. The percentage of each subfield with opacity was recorded and combined into a percentage of total opacity involvement for the eye. We used worst scores of cortical cataract during the 15-year observation period as quantitative traits. We also defined a binary trait of severe cortical cataract status: if the score indicated that ≥25% of the entire lens in either eye was affected we classified them as affected, whereas unaffected individuals were those with cortical scores<1% for the worse eye. The history of affection status during the 20 -year observation period from baseline (prevalent) and three follow-up (incident) phases of the study was reviewed to classify individuals into a binary classification, affected/unaffected, of cortical cataract, eliminating those with intermediate scores.

We adjusted for covariates that were previously identified by epidemiologic studies [38]–[42]. Risk factors included continuous variables such as age at baseline (years; *age*) and highest body mass index (*bmi*; height divided by square of weight), and ordinal variables such as gender (male = 1, female = 0), diabetes status (*dm*; never affected at any time point = 0, moderately affected = 1, and severely affected = 2), history of heavy alcohol intake (four or more alcoholic drinks per day; never = 0, past = 1, and current = 2), history of cigarette smoking, defined as more than 100 cigarettes smoked during the lifetime (*smoke*; never = 0, past smoker = 1, current smoker = 2) , and age-related macular degeneration affection score (*amd*; 15-step score) [43]. Each covariate was considered independently and with second order (polynomial) terms, in the analysis. We also evaluated interaction terms between covariates. Each phenotype was predicted by a different set of risk factors. We selected the final models for the quantitative and binary traits using stepwise linear and logistic regression models, respectively. The quantitative cortical cataract was predicted by *age* and *gender*, and the binary cortical cataract was predicted by *age* and *age^2^*. We used residuals from each final model as the initial trait in our association analysis.

#### The United Kingdom Twin Eye Study

The United Kingdom Twin Eye Study (UKTS) consists of a total of 506 white female twin pairs (226 monozygotic and 280 dizygotic), between ages of 49 and 79 from the UK Adult Twin Registry [5]. IRB approval was obtained from the St Thomas' Hospital Local Research Ethics Committee, and all subjects signed informed consents. The research followed the tenets of the Declaration of Helsinki. Similar to the BDES, the UKTS sample showed evidence for linkage on chromosome 1p36 (D1S2697; P = 0.001). A total of 219 participants were available with DNA and cataract phenotypes. Both individuals from 13 dizygotic twin pairs were included in analyses. As 23 pairs were monozygotic twins, the twin with the more severe cataract score or with DNA available was retained for analysis. Thus, the number of individuals in the final analysis was 185 individuals from 172 families.

In UKTS, cortical cataract scores were graded from 0 to 5 in decimalized steps, which could be converted to percent of the lens affected on multiplying by a factor of 20. In the digital grading procedure, photos taken from participants were stored using a digitized retroillumination camera system and analyzed using the Wilmer Automated Cortical Cataract Grading System [5]. Similar to the BDES sample, a binary variable for severe cortical cataract was defined: a cortical score greater than or equal to 25% as ‘affected’ and no sign of cortical cataract (score = 0) as ‘unaffected’.

To adjust for risk factors in the UKTS, the following covariates were tested: *age* (years), average alcohol intake from current and previous periods (units per week; *alcohol*), history of smoking (in the past or current; *smoke*), and average systolic blood pressure from initial and subsequent measurements (measured in mm Hg; *sysbp*). Diabetic status was not considered because of the large amount of missing data (percent missing = 52.3%). When testing significant covariates, we considered polynomial functions (e.g. age^2^) as well as first-order interactions between any two covariates. We selected the final models for the quantitative and binary traits using stepwise linear and logistic regression models, respectively. The final model for the quantitative cortical cataract trait consisted of *smoke*, *sysbp* ^2^, *interaction between age and bmi*, *interaction between smoke and bmi*, *interaction between smoke and alcohol*, and *interaction between sysbp and alcohol*. The binary severe cortical trait was predicted only by *age*, *age* ^2^, *smoke*, *sysbp* ^2^, *alcohol* ^2^, *interaction between age and bmi*, *interaction between age and alcohol*, *interaction between alcohol and bmi*, and *interaction between smoke and alcohol*. The residuals for the severe binary and quantitative cortical cataract traits were used in the association analyses.

#### Blue Mountains Eye Study

The Blue Mountains Eye Study (BMES) collected data in a suburban Australian population aged 49 years and older at baseline [44]. Ethics approval was obtained from the University of Sydney and Sydney West Area Human Research Ethics Committees, and written informed consent forms were collected from all study subjects; the research followed the tenets of the Declaration of Helsinki. BMES is unascertained for disease with the sampling frame based on postal codes in the region of the Blue Mountains area, west of Sydney. It has never been tested for association of age-related cataract with any locus [44].

All the definitions for cataract traits determined in the BMES followed the same grading protocol as that used in the BDES. Related individuals were excluded (N = 99) by only keeping the oldest individual from each family. The oldest individuals were selected because they were likely to bear the most amount of phenotypic information. A total of 1470 individuals were available with DNA and phenotypic data after removing related individuals. We considered as risk factors continuous variables such as age (years; *age*) and systolic blood pressure (measured in mm Hg; *sysbp*), and the ordinal variables gender (male = 1, female = 0), hypertension status (*hypt*; severely affected at any time point = 2, moderately affected = 1, and never affected = 0), history of heavy smoking, defined as more than 100 cigarettes smoked during the lifetime (*smoke*; never = 0, past = 1, and current = 2), and age-related macular degeneration affection score (*amd*; 15-step score). We selected the final models for the quantitative and binary traits using stepwise linear and logistic regression models, respectively. The quantitative cortical cataract score was predicted by *gender* and *age*. The binary severe cortical cataract trait was predicted by *smoke*, *sysbp*, *interaction between age and gender*, *interaction between age and sysbp*, and *interaction between sysbp and smoke*. We used residuals from each final model as the trait in our association analysis, for both the severe binary and quantitative cortical cataract trait.

### Re-sequencing

We selected 34 individuals for re-sequencing, 19 of whom had severe cortical cataract (worst cortical score ≥25% of lens involved), 15 were unaffected family members with no evidence of cataract formation (worst cortical score <1%) at ≥70 years of age, representing 18 families. Our rationale for choosing this set of individuals for re-sequencing was that they were previously shown to be linked to markers at 1p36 [13] and we hoped to find coding variants associated with disease, in addition to finding neutral variants.

To identify linked families, sib pairs from the SIBPAL program (version 5.3.1) in the candidate region (1p36) were ranked for linkage informativity [45]. For each sibling pair, a score based on the squared sib-pair difference and the estimated sib-pair marker allele sharing was computed:
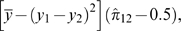
where 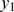 and 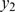 are the trait values for sibs 1 and 2, 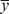 is the average of 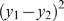 over the whole sample, 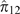 is the estimated mean allele sharing for the two sibs. This score is large (positive) either when the squared sib pair difference of the trait value is small and 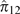 is large or when the squared sib-pair difference is large and 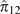 is small, which means the sibs are similarly alike, in terms of deviation from the mean, for both their traits and allele-sharing; otherwise the score will tend to be small (negative). Therefore, all the sibpairs with positive values were considered to be contributing to linkage.

All 17 *EPHA2* exons along with 50–100 base pairs of the surrounding intronic junctions were sequenced with the polymerase chain reaction (PCR) with primers designed using the PrimerSelect program (version 4.05) in the DNASTAR software (Table S1). For each amplicon, the PCR conditions for amplification are described in Table S1. After PCR, the product was purified using ExoSAP-IT enzyme (1×; 2 µl of the ExoSAP-IT enzyme with 10 µl PCR product for a final concentration of 5 ng/µl) and the product sizes were confirmed on a 1.5% agarose gel. The amplified PCR products along with primers were sent to McLab (San Francisco, CA) for sequencing. Electropherograms were examined using the ChromasPro program (version 1.4) and new variants not previously reported in the literature were confirmed by sequencing in the reverse direction.

### Single Nucleotide Polymorphism (SNP) Selection

We selected SNPs for full-scale genotyping via three strategies: re-sequencing, tag-based selection using the linkage disequilibrium (r^2^), and phylogenetic mining. Based on the results of re-sequencing, three rare coding variants and a common SNP were selected for follow up in the remaining individuals of the discovery data. For the tag-based approach, linkage disequilibrium (LD) in *EPHA2* was investigated by examining SNPs from 10 Kb upstream (+10 Kb) to 10 Kb downstream (−10 Kb) of the gene (16,323,419–16,355,151 bp) in HapMap data (CEU; Centre d'Etudes du Polymorphisme Humain; Utah residents with ancestry from northern and western Europe, HapMap Data, Data Rel 21 a/phaseII Jan07, on NCBI B36.3 assembly, dbSNP b130) using the HAPLOVIEW program (version 3.1). We selected at least one SNP in each block based on HapMap data to get four tagging SNPs.

Phylogenetic footprinting [46] and phylogenetic shadowing [47] were used for additional SNP selection. Under the assumption that non-coding regulatory regions are conserved through evolution, direct genotyping of non-coding conserved SNPs may increase power in chromosomal regions with low LD. We examined mouse-human sequences to maximize the number of inter-species conserved regions. The *EPHA2* sequences from human (*Homo sapiens*) and mouse (*Mus musculus*) were aligned using zPicture. We used window size width of ≥100 bp with ≥70% identity between human and mouse [48]. In the phylogenetic shadowing approach, using multiple sequence comparisons among primates, sequence elements (blocks) that did not change among primates are identified as regulatory elements [47]. We identified the non-coding conserved regions in *EPHA2* by comparing the human *EPHA2* gene as a reference with the orthologous sequences in two primates, the chimpanzee (*Pan troglodytes*) and the rhesus monkey (*Macaca mulatta*) using eShadow [47]. Within each conserved block one SNP with the highest minor allele frequency (MAF) was selected for genotyping.

Based on the hybrid selection strategy for SNPs, we first selected four non-redundant tag based SNPs that had MAF>5%. These SNPs were supplemented with 11 conserved non-coding SNPs identified through phylogenetic approaches. Finally, a set of intriguing coding and regulatory SNPs (N = 4) identified through re-sequencing were also genotyped. In total, 19 SNPs were genotyped in all three datasets, the BDES, UKTS, and BMES.

### SNP Genotyping and Quality Control Metrics

We used two different techniques, TaqMan and SNPlex assays, to interrogate SNPs. The genotyping procedure was performed according to the manufacturer's protocol. Clustering algorithms for genotype assignment used three quality control steps: (1) removing wells (individuals) with a significant number of outlier SNPs, (2) setting a SNP pass value by assigning a pass or fail status for each SNP, and (3) for each passing SNP, a confidence value was calculated (call rate). A confidence value of at least 95% was required to retain a genotype call. We further cleaned the data by removing individuals with low quality DNA across the SNPs (at least 90% of SNPs met the threshold quality value). We calculated the error rate and no-call rate using 5% replicate plating of DNA prior to these filtering steps. The average error rate and no-call rate for SNPlex genotyping was 0.7% and 3.9%, respectively. The average error rate and no-call rate for TaqMan genotyping was 0.3% and 3.7%, respectively. Cleaned data were used in association analyses.

All genotyped SNPs met Hardy-Weinberg equilibrium with a nominal P>0.05. We estimated allele frequencies using the FREQ program in S.A.G.E. (version 5.4.2), and 95% confidence intervals were reported (Table S2). The program estimates allele frequencies using a maximum likelihood formulation that assumes a random mating population. Standard errors are computed by numerical double differentiation of the log likelihood (Table S2). We also examined linkage disequilibrium (LD) blocks in each dataset using the HAPLOVIEW program (version 3.32).

### Statistical Methods

#### Association analysis for family data

All genotyped SNPs were tested for association using the ASSOC program in S.A.G.E. (version 5.3.1). The method in ASSOC is a maximum likelihood regression-based approach that allows appropriate handling of familial correlations, estimating as necessary variance components for *polygenic*, *marital*, *family*, and *sibling effects* as well as for *individual random effect*s, and that transforms both sides of the regression equation [49] (simultaneously estimating two transformation parameters) to remove skewness and kurtosis [27]. The model for the *i^th^* pedigree member is *h(y_i_) = h(f_i_)+ε_i_*, where *y_i_* is the trait value, *f_i_ = α+βg_i_*, *g_i_* is the genetic marker value, *ε_i_* is the sum of the random effects, and *h* is the George-Elston generalized modulus power transformation [27]. The George-Elston transformation is defined as
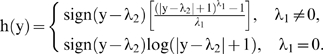
This transformation involves both power (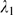) and shift (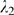) parameters that are simultaneously estimated, together with the other parameters of the model, by maximum likelihood under the assumption of multivariate normality across family members. Because the transformation is applied to both the trait value (y) and its expectation *f*, for quantitative traits the regression coefficients (β) are median unbiased estimates [49] to the extent that the model fits. In the case of binary traits, because the quantitative residuals (y) were obtained from logistic regression, the estimated regression coefficients (β) are not meaningful with respect to the magnitude of the effect, though they are so with respect to their statistical significance. Three genetic models (*g*), additive, dominant, and recessive with respect to a reference allele, were assessed at each SNP. The minor allele was selected as the reference allele. For example, a SNP in an additive model was coded as 0, 1, or 2 according to the number of reference alleles, whereas it was coded as 0 or 1 in a dominant model according to the absence or presence of the reference allele.

To find the best model for the familial correlation structure, each familial variance component was tested in the model by itself and in combination with others. The most significant, but parsimonious, model was retained as the “null” model against which was contrasted models containing the SNPs as covariates. For the two family datasets BDES and UKTS, the variance components for *sibling* and *random effects*, along with a genetic model for a SNP, were included in the final analysis. For each SNP, the baseline model without the SNP covariate and an alternate model with the SNP covariate were contrasted. The likelihood was maximized with respect to the null model containing the variance components and the baseline parameter α, and the alternate model containing the same variance components and both α and β. We performed likelihood ratio tests by comparing twice the difference between two log likelihoods with a chi-square distribution, and Wald tests by dividing each estimate of β by its standard error, estimated by numerical double differentiation of the log likelihood, and comparing the quotient to a standard normal distribution. Thus we obtained two P values that would be asymptotically identical.

We examined the residuals ([*h(y_i_)−h(α+βg_i_)*]) in a final model that included the most significant SNP by plotting a normal quantile-quantile (normal Q-Q) plot. Regardless of the distribution of *y*, normality of the residuals is required for the association result to be valid. We also conducted association analyses on the normalized traits, forcing the marginal distribution of the analyzed trait to be about normally distributed under the null hypothesis, by taking the inverse standard normal transformation of the empirical quantile that was obtained from the formula [*r(y)−1/3*] divided by *(n+1/3)*, where *r(y)* is the rank of *y* [50]. To evaluate the ability of the George-Elston transformation to achieve normality, we compared residuals without transformation to those with the George-Elston transformation, and to those with the George-Elston transformation when the initial trait was inverse normal transformed. We examined the distributions of residuals from the final models using the quantitative cortical cataract trait in the BDES and in UKTS. Since the estimates (β-coefficients) for the quantitative trait, after the George-Elston transformation is applied to both sides of the regression equation, are median unbiased estimates, we report these estimates as our effects that measure net change, after adjusting for other covariates, due to a dominant or recessive genotype, or per copy of a reference allele (additive model). The estimate measures the net percentage change in cataract score ranging from 0 to 100%.

For the severe binary cortical cataract trait, as the George-Elston transformation was not sufficient to obtain normality in both family datasets, we report only P values from inverse normal transformed traits. For comparability, P values from both the quantitative and binary traits in the main text were from inverse normal transformed traits, and estimates for the quantitative cortical cataract were only reported for quantitative traits without inverse normal transformation. We also report P values from the likelihood ratio tests and Wald tests of the inverse normal transformed quantitative and binary traits.

#### Association analysis for unrelated data

For the unrelated BMES data, we performed ordinary least squares regression of quantitative traits on allele dosage as implemented in the PLINK program (version 1.0.1) [51]. We used Wald tests based on t-distributions, and regression coefficients (β-coefficients) and asymptotic P values are reported. We performed the Cochran-Armitage (additive) trend test using an age-matched (age at least 70 years) case-control set, comprising cases with cortical score ≥25% and controls with cortical score <1%. The PLINK program performs a chi-square test for association on the fourfold allele table, and reports asymptotic P values and the estimated odds ratios for the minor allele. We also report the 95% confidence intervals for the odds ratios.

After single SNP association tests, for which the P values were moderate, we conducted haplotype-based association tests in the unrelated data in order to explore the improvement in significance. Using the five most significantly associated SNPs (rs7548209, rs3754334, rs3768293, rs6603867 and rs6678616), moving windows of different sizes (sizes 2 to 5) were applied using the PLINK program (version 1.0.1). The PLINK program probabilistically infers haplotypes for each individual based on the standard expectation-maximization phasing [51]. We tested the association of haplotypes with the quantitative cortical cataract trait and obtained regression coefficients (β), t-statistics, and asymptotic significance values for the coefficients.

#### Heterogeneity test and meta-analysis

We tested heterogeneity of effect sizes for SNP associations using the Q test assuming a random-effects model, which estimates the variability of the population effect size across studies [52]. In this model, we assume population effect sizes (*β_i_*) are heterogeneous among studies and follow a normal distribution with mean 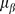 and variance 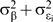. This assumes the individual sampling errors are independent and normally distributed with variance 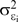. This assumption is valid, especially when residuals from the final model are normally distributed. The Q statistic was constructed using the formula, 
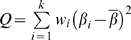
, where 
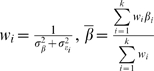
, and *k* is the number of studies. Under the null hypothesis (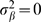) and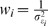, the Q statistic asymptotically follows a chi-squared distribution with k-1 degree of freedom. The null hypothesis will be rejected at the 5% level when the observed test statistic exceeds the *95^th^* percentile of a chi-squared distribution. We used effect sizes (*β_i_*) and sampling variances (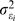, squared standard errors of the estimates) to calculate the Q statistic. Asymptotically, when the sample sizes of all *k* studies are sufficiently large, the Q statistic follows a chi-squared distribution.

We conducted meta-analysis using the METAL program, which calculates an overall z-statistic and P value from the weighted average of the individual statistics, accounting for sample size by calculating the square-root of the number of individuals examined in each sample (http://csg.sph.umich.edu/). We report N, total number of analyzable valid individuals, Z-scores, and overall P values in the combined dataset.

## Supporting Information

Figure S1 Morphological, histochemical and biochemical characterization of wild type and mutant mouse lens. (A) H&E sections of lenses from eight-month- and two-week-old mice. Note abnormal structure of the *Epha2* homozygous knockout lens. Scale bars: 1 mm for left two panels; 10 µm for right two panels. (B) Anterior view of an intact *Epha2−/−* lens stained with X-gal to detect LacZ reporter gene expression. With 3-hour short-term staining, expression in epithelial cells on lens surface but not in deeper fiber cells was detected. (C) X-gal staining of frozen sagittal section showing LacZ reporter expression pattern. Inset is shown in (D). Scale bars: 1 mm for B and C; 10 µm for D. (E) Staining of an *Epha2−/−* lens using an antibody recognizing the ectodomain of EPHA2. Because part of the EPHA2 ectodomain was fused to LacZ reporter gene, the staining pattern is similar to that of X-gal as in D; both were trapped in the secretory vesicles. F) EPHA2 protein expression in the isolated lens cortex and nuclei. Note the age-dependent decrease in EPHA2 expression. Same amounts of protein extracts were loaded as indicated by Ponceau S staining.
(2.87 MB TIF)

Figure S2 EPHA2 expression in human lens. (A) Lysates were prepared from two 56 and 60 year old human lenses and blotted for EPHA2. (B–D) EPHA2 staining of human lens: Images from sagittal sections at anterior (B) and posterior (C) regions and coronal section near the equator (D) are shown. (E) Negative control without primary antibody. Scale bars: 5 µm.
(4.16 MB TIF)

Figure S3 Crystallin expression and activation status of Akt and ERK1/2 kinases in wild type and mutant mice. (A) *Epha2* deletion did not cause significant changes in crystallin expression until development of mature cataract. Each lens from the indicated age was extracted with RIPA buffer first. After centrifugation, the supernatant was collected as soluble fraction. The pellet was directly dissolved in protein gel loading solution containing 2% SDS. The soluble and insoluble fractions were separated and blotted with the indicated antibodies. (B) *Epha2* deletion did not significantly alter the ERK1/2 and Akt kinase activities. Total lens proteins were extracted with RIPA buffer and subject to immunoblot with the indicated antibodies.
(1.29 MB TIF)

Figure S4 Normal Quantile-Quantile (Q-Q) plots from residuals under the dominant model. The first column is the residuals from the initial trait without any transformations (NOTF), the second column is the residuals from the initial trait with the George-Elston transformation (GETF), and the third column is the inverse normal transformed adjusted data with the George-Elston transformation (INV-GETF). (A–B) Normal Q-Q plots from residuals of the Arg721Gln variant were plotted for the BDES. (A) The plots were drawn for cortical cataract. (B) The plots were drawn for severe cortical cataract. (C–D) Normal Q-Q plots from residuals of rs7548209 were plotted for the UKTS. (C) The plots were drawn for cortical cataract. (D) The plots were drawn for severe cortical cataract.
(0.76 MB TIF)

Figure S5 Retroillumination images of normal human lens vs. lens with cortical cataract. A normal human lens. (A) compared with cortical cataract lens from the Beaver Dams Eye Studies (BDES) (B) and Blue Mountain Eye Studies (BMES) (C). (D) A panel of retroillumination images from UKTS going from normal in the lower right corner to varying degrees of cortical cataract. The lens in the bottom left panel is the most severe.
(9.61 MB TIF)

Figure S6 Plots of linkage disequilibrium (LD) for *EPHA2*. Each LD plot is based on correlation coefficient of LD (r2) in the HapMap CEU (A), BDES (B), UKTS (C), and BMES (D) datasets.
(2.84 MB TIF)

Table S1 Exonic primers and their amplification conditions for *EPHA2* gene re-sequencing.
(0.05 MB DOC)

Table S2 Completeness of SNP genotyping and other quality metrics.
(0.07 MB DOC)

Table S3 Single SNP association using ASSOC in the BDES family data.
(0.14 MB DOC)

Table S4 Single SNP association using ASSOC in the UKTS family data.
(0.14 MB DOC)

Table S5 Association results using PLINK in the BMES unrelated data. (A) P values from single SNP association under the dominant model. (B) P values from single SNP trend test for the severe cases and controls with age at least 70 years in the BMES. (C) Haplotype association for the binary and quantitative cortical cataract trait in BMES.
(0.10 MB DOC)

Table S6 Heterogeneity test and meta-analysis using METAL (A to D). Three genetic models (Add: additive, Dom: dominant, and Rec: recessive) were tested for each SNP. QE-P: P values associated with the Q statistic; Zscore: Z-score from meta-analysis; N: the total number of individuals in the meta-analysis; Meta-P: P values from meta-analysis. (A) Heterogeneity test (Q test) and meta-analysis for the quantitative cortical cataract from the BDES and UKTS. (B) Heterogeneity test (Q test) and meta-analysis for severe cortical cataract from the BDES and UKTS. (C) Heterogeneity test (Q test) and meta-analysis from all three datasets (BDES, UKTS, and BMES) under the dominant model.
(0.23 MB DOC)
