# Supplementary figures and images for: EPHA2 Is Associated with Age-Related Cortical Cataract in Mice and Humans
Source: PLoS Genet. 2009 Jul 31;5(7):e1000584. doi: 10.1371/journal.pgen.1000584 (PMC2712078; doi:10.1371/journal.pgen.1000584)

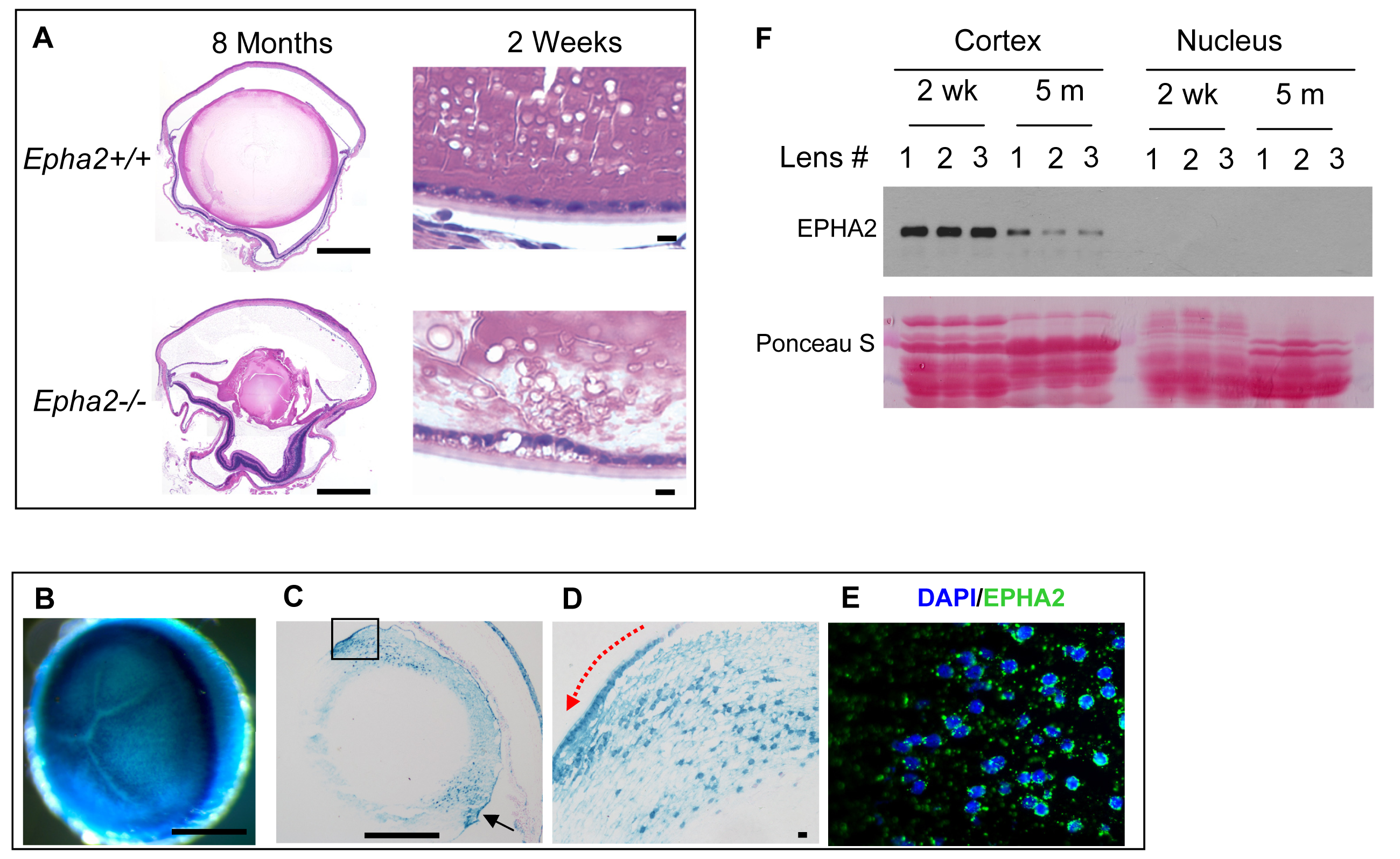

Supplement: Figure S1 — Morphological, histochemical and biochemical characterization of wild type and mutant mouse lens. (A) H&E sections of lenses from eight-month- and two-week-old mice. Note abnormal structure of the Epha2 homozygous knockout lens. Scale bars: 1 mm for left two panels; 10 µm for right two panels. (B) Anterior view of an intact Epha2−/− lens stained with X-gal to detect LacZ reporter gene expression. With 3-hour short-term staining, expression in epithelial cells on lens surface but not in deeper fiber cells was detected. (C) X-gal staining of frozen sagittal section showing LacZ reporter expression pattern. Inset is shown in (D). Scale bars: 1 mm for B and C; 10 µm for D. (E) Staining of an Epha2−/− lens using an antibody recognizing the ectodomain of EPHA2. Because part of the EPHA2 ectodomain was fused to LacZ reporter gene, the staining pattern is similar to that of X-gal as in D; both were trapped in the secretory vesicles. F) EPHA2 protein expression in the isolated lens cortex and nuclei. Note the age-dependent decrease in EPHA2 expression. Same amounts of protein extracts were loaded as indicated by Ponceau S staining. (2.87 MB TIF) [file pgen.1000584.s001.tif]

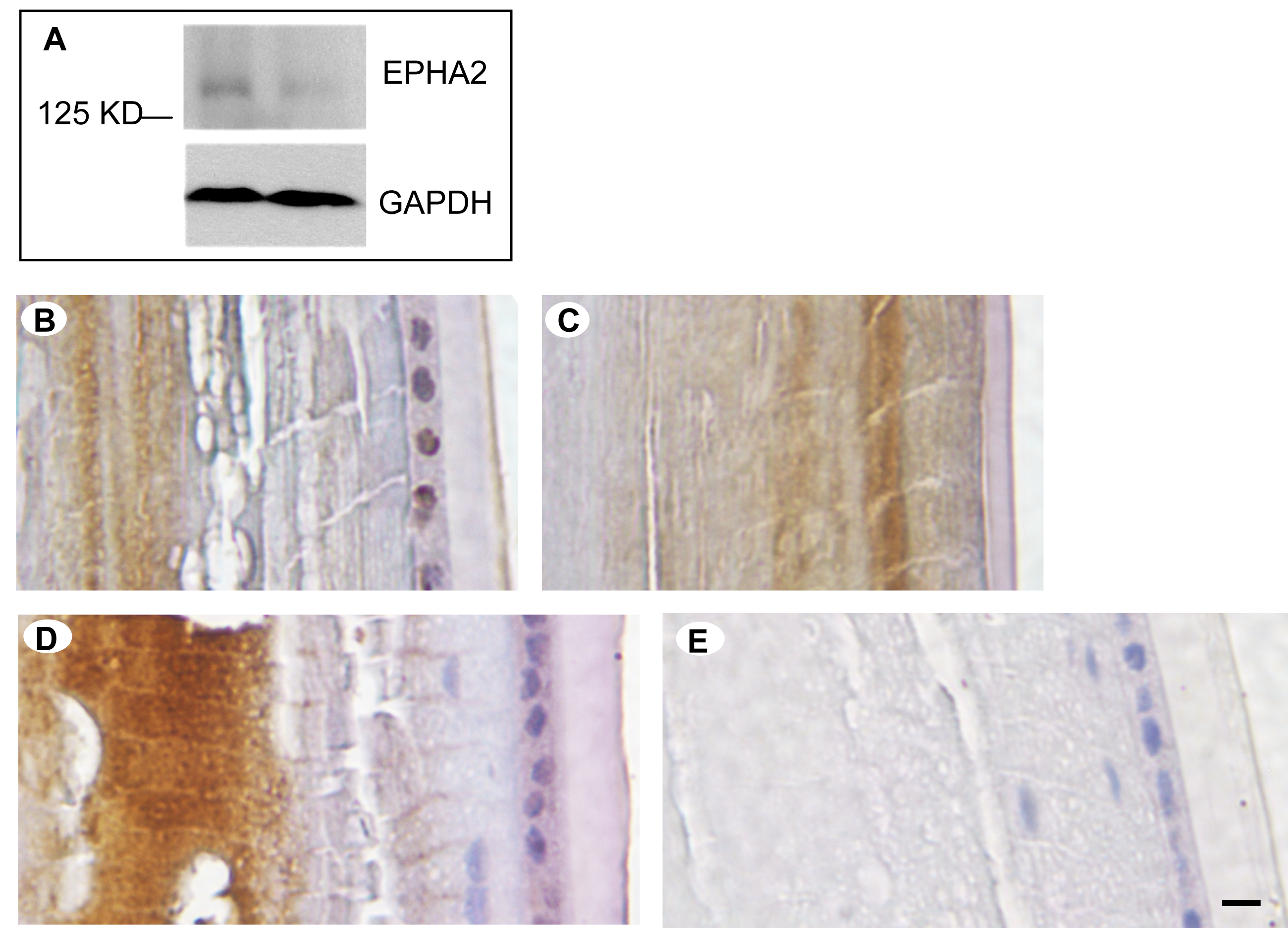

Supplement: Figure S2 — EPHA2 expression in human lens. (A) Lysates were prepared from two 56 and 60 year old human lenses and blotted for EPHA2. (B–D) EPHA2 staining of human lens: Images from sagittal sections at anterior (B) and posterior (C) regions and coronal section near the equator (D) are shown. (E) Negative control without primary antibody. Scale bars: 5 µm. (4.16 MB TIF) [file pgen.1000584.s002.tif]

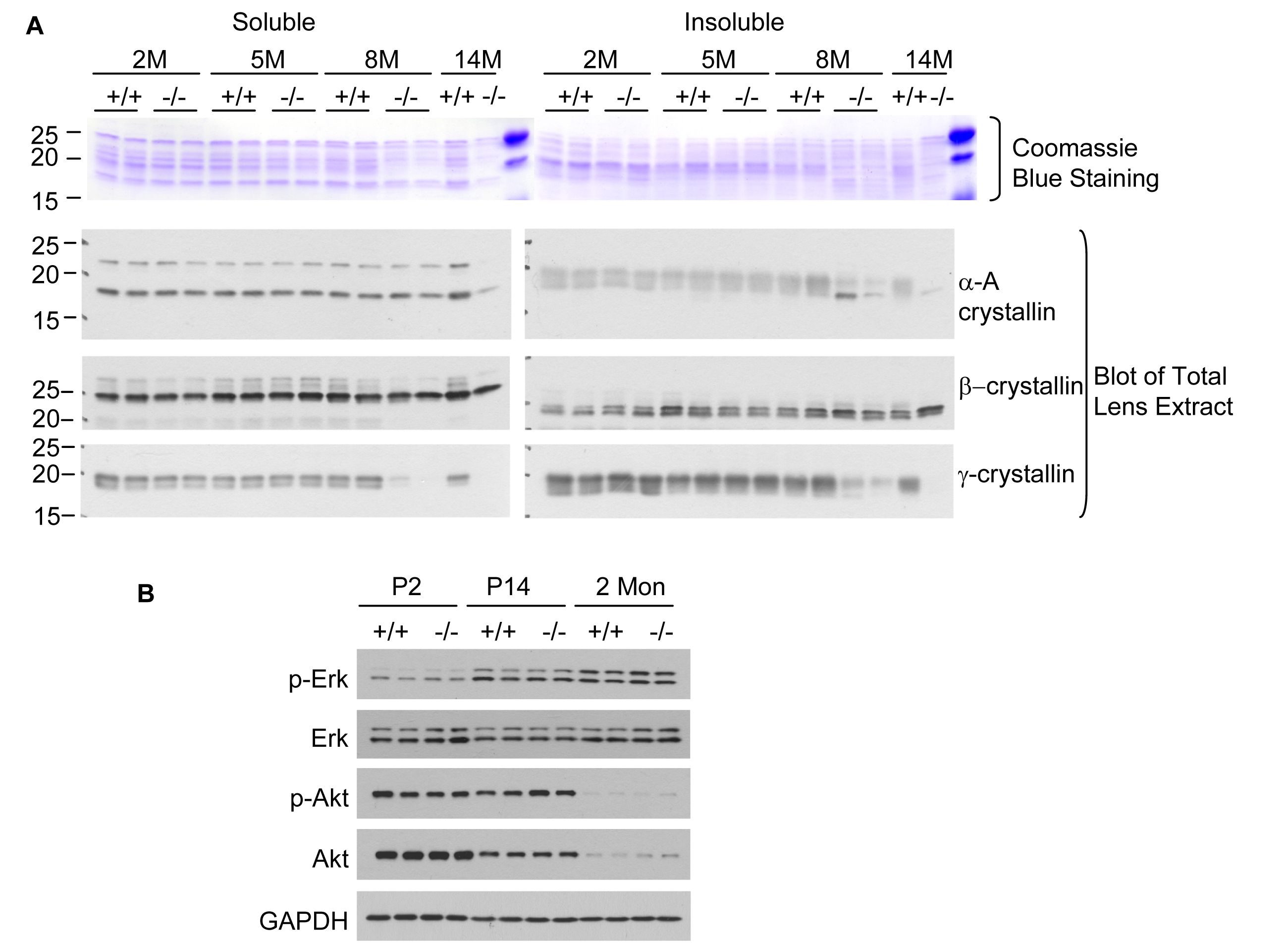

Supplement: Figure S3 — Crystallin expression and activation status of Akt and ERK1/2 kinases in wild type and mutant mice. (A) Epha2 deletion did not cause significant changes in crystallin expression until development of mature cataract. Each lens from the indicated age was extracted with RIPA buffer first. After centrifugation, the supernatant was collected as soluble fraction. The pellet was directly dissolved in protein gel loading solution containing 2% SDS. The soluble and insoluble fractions were separated and blotted with the indicated antibodies. (B) Epha2 deletion did not significantly alter the ERK1/2 and Akt kinase activities. Total lens proteins were extracted with RIPA buffer and subject to immunoblot with the indicated antibodies. (1.29 MB TIF) [file pgen.1000584.s003.tif]

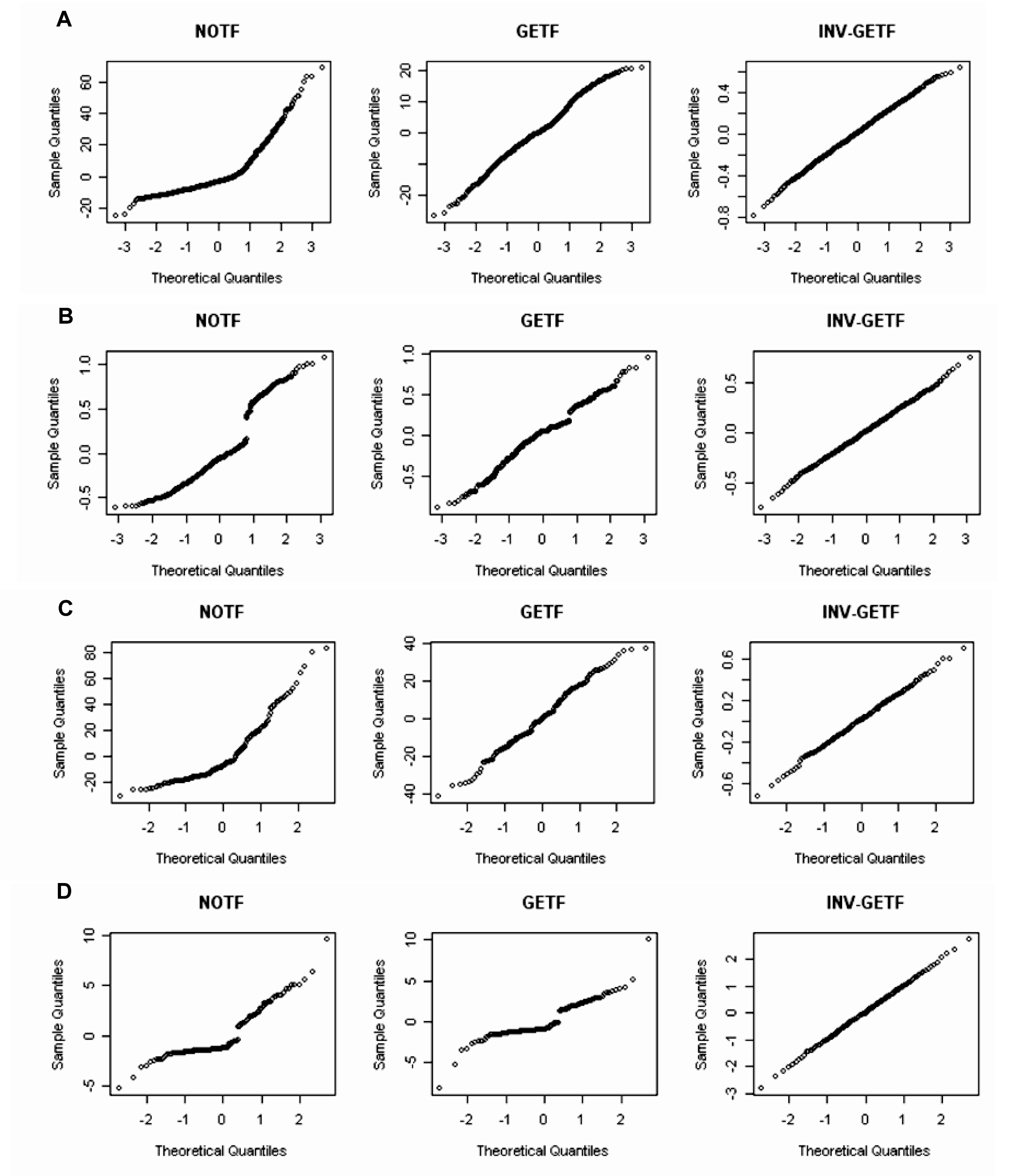

Supplement: Figure S4 — Normal Quantile-Quantile (Q-Q) plots from residuals under the dominant model. The first column is the residuals from the initial trait without any transformations (NOTF), the second column is the residuals from the initial trait with the George-Elston transformation (GETF), and the third column is the inverse normal transformed adjusted data with the George-Elston transformation (INV-GETF). (A–B) Normal Q-Q plots from residuals of the Arg721Gln variant were plotted for the BDES. (A) The plots were drawn for cortical cataract. (B) The plots were drawn for severe cortical cataract. (C–D) Normal Q-Q plots from residuals of rs7548209 were plotted for the UKTS. (C) The plots were drawn for cortical cataract. (D) The plots were drawn for severe cortical cataract. (0.76 MB TIF) [file pgen.1000584.s004.tif]

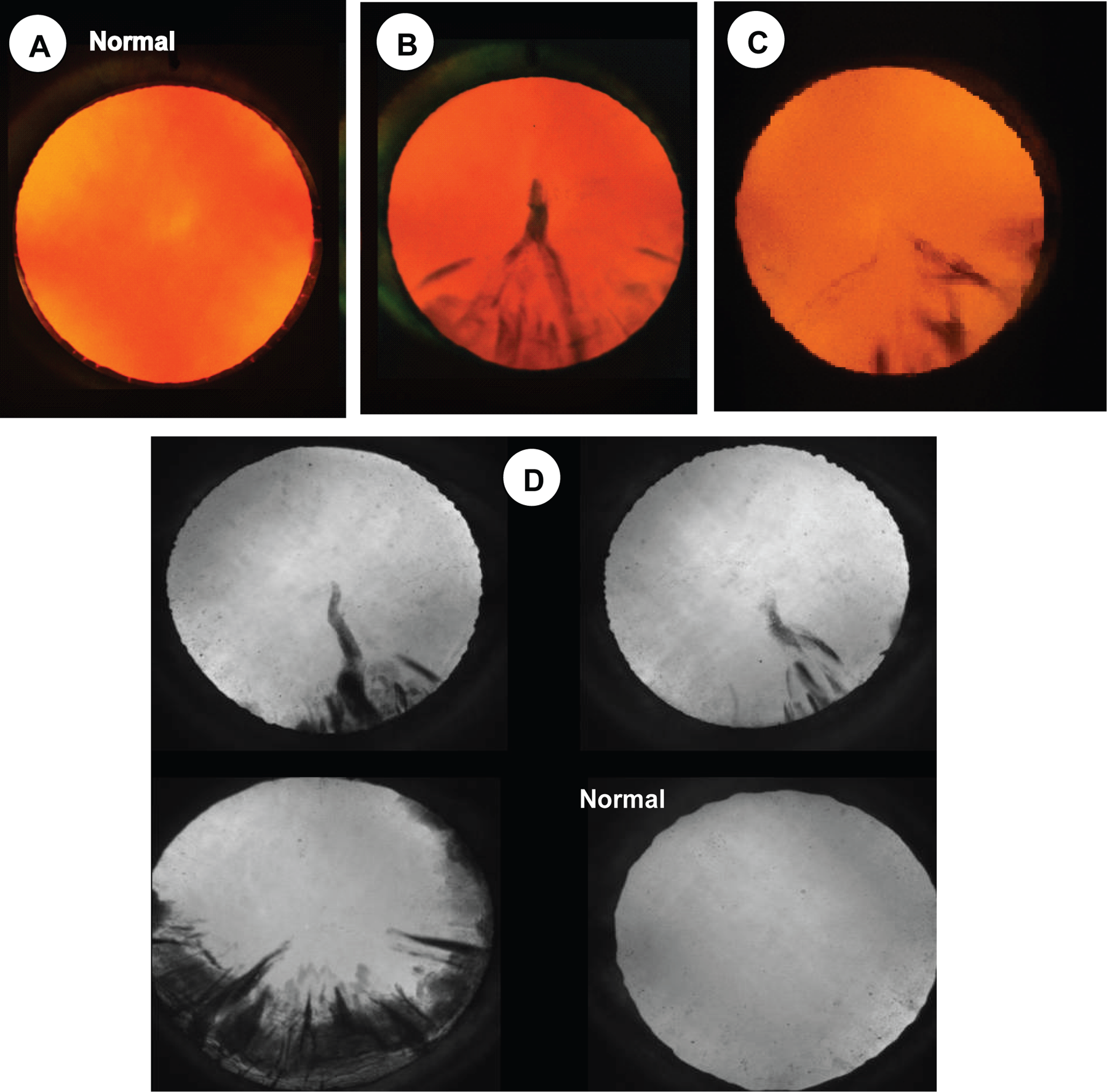

Supplement: Figure S5 — Retroillumination images of normal human lens vs. lens with cortical cataract. A normal human lens. (A) compared with cortical cataract lens from the Beaver Dams Eye Studies (BDES) (B) and Blue Mountain Eye Studies (BMES) (C). (D) A panel of retroillumination images from UKTS going from normal in the lower right corner to varying degrees of cortical cataract. The lens in the bottom left panel is the most severe. (9.61 MB TIF) [file pgen.1000584.s005.tif]

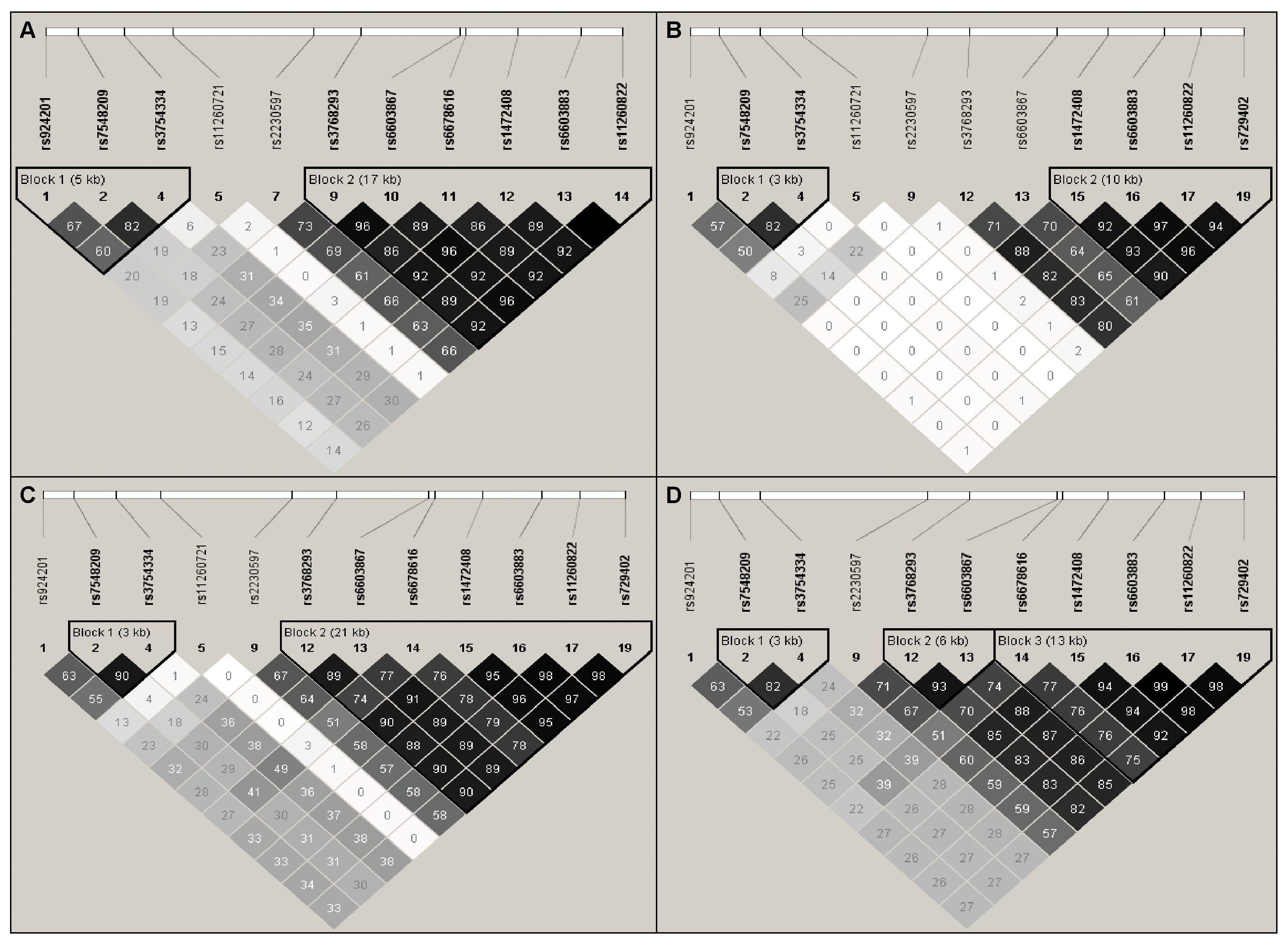

Supplement: Figure S6 — Plots of linkage disequilibrium (LD) for EPHA2. Each LD plot is based on correlation coefficient of LD (r2) in the HapMap CEU (A), BDES (B), UKTS (C), and BMES (D) datasets. (2.84 MB TIF) [file pgen.1000584.s006.tif]
