# Supplementary material for: EPHA2 Is Associated with Age-Related Cortical Cataract in Mice and Humans
Source: PLoS Genet. 2009 Jul 31;5(7):e1000584. doi: 10.1371/journal.pgen.1000584 (PMC2712078; doi:10.1371/journal.pgen.1000584)
Supplement: Table S1 — Exonic primers and their amplification conditions for EPHA2 gene re-sequencing. (0.05 MB DOC) [file pgen.1000584.s007.doc]

Table S1. Exonic primers and their amplification conditions for *EPHA2* gene re-sequencing

| Primer | F Primer Sequence | R Primer Sequence | Size (bp) | %GC (F-R) |
| --- | --- | --- | --- | --- |
| Exon 1 | GCCCCTTTAAAGACATTCC | TTATTCTCCGGAGCCCCTATGA | 503 | 47.4-50.0 |
| Exon 2 | GGGTTCCATCCTTTTTCTG | ACCACCCATCCACCTCTTG | 597 | 47.4-57.9 |
| Exon 3A | GTAGAACCCGGAGAGCACGAACT | GCTCTGATGCACCTTCCCTGGC | 630 | 56.5-63.6 |
| Exon 3B | GATTGACACCATTGCGCCCGAT | ATCCTTCCCAGACATCAGTTTCCT | 558 | 54.5-45.8 |
| Exon 4 | TGCATTTTTCCATCACAAGACA | GAGCCCCACATGAACTACA | 656 | 36.4-52.6 |
| Exon 5 | CCTGCTCGCCTGGATTTTT | AGCAGAGCAAGTGGTTAAAGGCA | 697 | 52.6-47.8 |
| Exon 6 | AGTTCAAAGCTGTAGTGGGC | GTTACATCTCCCAAGGCAGCACAG | 474 | 50.0-54.2 |
| Exon 7 | ATGCATTTAGTGGTTCTGA | TTTGAGCCCCACATACCTGCA | 623 | 36.8-52.4 |
| Exon 8 | CTTTGAGCCCCACATACCT | GTCATTACCTCTGCTCTTGTTGTT | 529 | 52.6-41.7 |
| Exon 9 | TGGACACGCAGGCAGGAAT | CGTGACCTTCTCCTCTGACTCCA | 557 | 57.9-56.5 |
| Exon 10 | CCCCGGAGGACGTTTACTTCT | GCTGAAGACATCCTCGGGG | 567 | 57.1-63.2 |
| Exon 11 | AAGGTGATCGGAGCAGGTGAGGTT | CTGTCCTCAATACCTGTGCCCTCC | 823 | 54.2-58.3 |
| Exon 12 | TGTGCCCTCCTCCCCAATACCTGA | AGTGTAAGTTGGGGAAGGGGACC | 638 | 58.3-56.5 |
| Exon 13 | TAAGGATGTGGGTTGTAGGGG | CGTGTGGAGCTTTGGCATTGT | 597 | 52.4-52.4 |
| Exon 14 | ACCAGTGTAAGTTGGGGAAGGG | ATTTTTAGTAGAGACGGGGTTTCA | 669 | 54.5-37.5 |
| Exon 15 | CTGCCGTGAGGTGTGATAGC | CTTAGACTGGACACGATGGCCC | 641 | 60.0-59.1 |
| Exon 16 | AGATAAGGAGACGGAGGCACGGGG | ATTGGTAGAGAAACAGGCTGA | 769 | 62.5-42.9 |
| Exon 17A | TCTCCACCCTCCTCCTCTGCCT | TGAGCACTTAGCAGGCACCGC | 474 | 63.6-61.9 |
| Exon 17B | ACAGCGACATCAAGAGGA | TACTTTGTGGAGAGAATGTGT | 714 | 50.0-38.1 |
| Exon 17C | ACTGTGAACTTGACTGGGTGA | TAAACAGAGATTCGTGCACAAGGC | 571 | 47.6-45.8 |
