# Supplementary material for: EPHA2 Is Associated with Age-Related Cortical Cataract in Mice and Humans
Source: PLoS Genet. 2009 Jul 31;5(7):e1000584. doi: 10.1371/journal.pgen.1000584 (PMC2712078; doi:10.1371/journal.pgen.1000584)
Supplement: Table S2 — Completeness of SNP genotyping and other quality metrics. (0.07 MB DOC) [file pgen.1000584.s008.doc]

Table S2. Completeness of SNP genotyping and other quality metrics

| SNP^a^ | RA^b^ |  | BDES^c^ | |  | UKTS | |  | BMES | |
| --- | --- | --- | --- | --- | --- | --- | --- | --- | --- | --- |
|  |  |  | %Geno | RAF (95%CI) |  | %Geno | RAF (95%CI) |  | %Geno | RAF (95%CI) |
| rs924201 | A |  | 99.1 | 0.396 (0.373, 0.420) |  | 100.0 | 0.599 (0.55, 0.648) |  | 99.3 | 0.623 (0.602,0.643) |
| rs7548209 | C |  | 98.7 | 0.331 (0.308, 0.353) |  | 100.0 | 0.338 (0.29, 0.386) |  | 100.0 | 0.312 (0.293,0.332) |
| rs1803527 | C |  | 99.2 | 0.301 (0.279, 0.322) |  | 96.5 | 0.300 (0.248, 0.353) |  | 99.3 | 0.288 (0.272,0.305) |
| rs3754334 | A |  | 99.8 | 0.021 (0.015, 0.028) |  | 99.7 | 0.000 (0.000, 0.011^d^) |  | 100.0 | 0.034 (0.026,0.041) |
| rs11260721 | G |  | 99.7 | 0.151 (0.134, 0.167) |  | 99.9 | 0.121 (0.088, 0.154) |  | 100.0 | 0.098 (0.085,0.111) |
| Ile779Ile | T |  | 99.5 | 0.028 (0.020, 0.035) |  | 98.5 | 0.026 (0.008, 0.044) |  | 99.3 | 0.012 (0.008,0.016) |
| Arg721Gln | A |  | 95.0 | 0.006 (0.002, 0.009) |  | 96.0 | 0.000 (0.000, 0.011^d^) |  | 99.3 | 0.002 (0.000,0.004) |
| rs13375644 | A |  | 99.8 | 0.088 (0.075, 0.101) |  | 100.0 | 0.089 (0.061, 0.118) |  | 100.0 | 0.052 (0.043,0.062) |
| rs2230597 | A |  | 99.7 | 0.427 (0.403, 0.450) |  | 97.5 | 0.380 (0.327, 0.434) |  | 100.0 | 0.401 (0.383,0.418) |
| Ser277Leu | T |  | 97.2 | 0.005 (0.002, 0.008) |  | 96.7 | 0.007 (0.000, 0.016) |  | 97.3 | 0.006 (0.003,0.009) |
| rs11260745 | G |  | 71.7 | 0.084 (0.069, 0.098) |  | 99.9 | 0.088 (0.059, 0.117) |  | 96.6 | 0.053 (0.043,0.062) |
| rs3768293 | G |  | 99.8 | 0.614 (0.591, 0.637) |  | 99.9 | 0.383 (0.328, 0.439) |  | 100.0 | 0.408 (0.390,0.426) |
| rs6603867 | C |  | 97.5 | 0.643 (0.620, 0.667) |  | 99.9 | 0.604 (0.554, 0.654) |  | 99.3 | 0.606 (0.588,0.624) |
| rs6678616 | A |  | 88.4 | 0.251 (0.229, 0.272) |  | 93.7 | 0.346 (0.298, 0.394) |  | 100.0 | 0.343 (0.322,0.364) |
| rs1472408 | T |  | 100.0 | 0.617 (0.594, 0.641) |  | 100.0 | 0.602 (0.553, 0.651) |  | 100.0 | 0.591 (0.570,0.612) |
| rs6603883 | T |  | 99.2 | 0.377 (0.353, 0.400) |  | 100.0 | 0.393 (0.343, 0.442) |  | 100.0 | 0.403 (0.382,0.424) |
| rs11260822 | A |  | 99.7 | 0.614 (0.590, 0.638) |  | 100.0 | 0.611 (0.561, 0.660) |  | 100.0 | 0.597 (0.576,0.618) |
| rs904106 | T |  | 100.0 | 0.047 (0.037, 0.057) |  | 99.6 | 0.053 (0.026, 0.080) |  | 89.1 | 0.051 (0.043,0.059) |
| rs729402 | G |  | 99.5 | 0.625 (0.601, 0.648) |  | 99.9 | 0.613 (0.564, 0.663) |  | 99.3 | 0.596 (0.575,0.617) |

^a^ Hardy-Weinberg Disequilibrium P values in all SNPs were greater than 0.05.

^b^ RA: reference alleles.

^c^ % Geno: genotyping completion; RAF: reference allele frequencies that were estimated using maximum likelihood by assuming the pedigrees are randomly ascertained; 95% CI: 95% confidence interval of frequencies.

^d^ Upper 95% limit for a sample size around 300.
