# Supplementary material for: EPHA2 Is Associated with Age-Related Cortical Cataract in Mice and Humans
Source: PLoS Genet. 2009 Jul 31;5(7):e1000584. doi: 10.1371/journal.pgen.1000584 (PMC2712078; doi:10.1371/journal.pgen.1000584)
Supplement: Table S3 — Single SNP association using ASSOC in the BDES family data. (0.14 MB DOC) [file pgen.1000584.s009.doc]

Table S3. Single SNP association using ASSOC in the BDES family data

| SNP | Model^a^ |  | Severe Cortical^b^ | |  | Cortical^c^ | | | |
| --- | --- | --- | --- | --- | --- | --- | --- | --- | --- |
|  |  |  | INV-LP | INV-WP |  | GE-β | GE-LP | INV-LP | INV-WP |
| rs924201 | Add |  | 0.0032 | 0.0031 |  | 0.34 | 0.1119 | 0.1663 | 0.1713 |
|  | Dom |  | 2 x 10^-4^ | 2 x 10^-4^ |  | 0.42 | 0.1457 | 0.1666 | 0.1668 |
|  | Rec |  | 0.7205 | 0.7202 |  | 0.45 | 0.2993 | 0.4632 | 0.4792 |
| rs7548209 | Add |  | 1 x 10^-4^ | 9 x 10^-5^ |  | 0.18 | 0.4302 | 0.4499 | 0.4494 |
|  | Dom |  | 4 x 10^-6^ | 4 x 10^-6^ |  | 0.21 | 0.4616 | 0.4516 | 0.4517 |
|  | Rec |  | 0.8031 | 0.8029 |  | 0.26 | 0.641 | 0.7216 | 0.721 |
| rs1803527 | Add |  | 0.0139 | 0.0134 |  | 0.12 | 0.8683 | 0.9507 | 0.9507 |
|  | Dom |  | 0.0139 | 0.0134 |  | 0.12 | 0.8683 | 0.9507 | 0.9507 |
|  | Rec |  | NA | NA |  | NA | NA | NA | NA |
| rs3754334 | Add |  | 0.0158 | 0.0158 |  | 0.26 | 0.2527 | 0.2635 | 0.2627 |
|  | Dom |  | 0.0225 | 0.0229 |  | 0.22 | 0.4228 | 0.3935 | 0.3935 |
|  | Rec |  | 0.1921 | 0.1934 |  | 0.73 | 0.2141 | 0.2898 | 0.286 |
| rs11260721 | Add |  | 5 x 10^-4^ | 5 x 10^-4^ |  | 0.69 | 0.0244 | 0.0154 | 0.0154 |
|  | Dom |  | 1 x 10^-4^ | 1 x 10^-4^ |  | 0.75 | 0.0221 | 0.0129 | 0.0128 |
|  | Rec |  | 0.3642 | 0.368 |  | 0.66 | 0.662 | 0.704 | 0.7039 |
| Ile779Ile | Add |  | 0.7634 | 0.7637 |  | -0.88 | 0.1579 | 0.0949 | 0.0974 |
|  | Dom |  | 0.5583 | 0.5592 |  | -1.08 | 0.1039 | 0.0659 | 0.0682 |
|  | Rec |  | 0.182 | 0.1855 |  | 2.54 | 0.5252 | 0.6905 | 0.6942 |
| Arg721Gln | Add |  | 6 x 10^-5^ | 8 x 10^-5^ |  | 9.20 | 2 x 10^-8^ | 2 x 10^-8^ | 8 x 10^-8^ |
|  | Dom |  | 6 x 10^-5^ | 8 x 10^-5^ |  | 9.20 | 2 x 10^-8^ | 2 x 10^-8^ | 2 x 10^-8^ |
|  | Rec |  | NA | NA |  | NA | NA | NA | NA |
| rs13375644 | Add |  | 0.2513 | 0.2491 |  | 0.18 | 0.6475 | 0.714 | 0.7137 |
|  | Dom |  | 0.1111 | 0.109 |  | 0.19 | 0.6482 | 0.7094 | 0.709 |
|  | Rec |  | 0.0025 | 0.0045 |  | 0.27 | 0.9125 | 0.9625 | 0.9624 |
| rs2230597 | Add |  | 0.0363 | 0.0362 |  | 0.44 | 0.0304 | 0.0234 | 0.0229 |
|  | Dom |  | 0.3718 | 0.3721 |  | 0.35 | 0.2409 | 0.2067 | 0.207 |
|  | Rec |  | 0.0064 | 0.0061 |  | 0.99 | 0.0103 | 0.0081 | 0.0084 |
| Ser277Leu | Add |  | 0.0483 | 0.0512 |  | -3.25 | 0.0462 | 0.0399 | 0.0454 |
|  | Dom |  | 0.0483 | 0.0512 |  | -3.25 | 0.0462 | 0.0399 | 0.0454 |
|  | Rec |  | NA | NA |  | NA | NA | NA | NA |
| rs11260745 | Add |  | 0.9099 | 0.8024 |  | -0.19 | 0.6712 | 0.6758 | 0.4994 |
|  | Dom |  | 0.9697 | 0.9697 |  | -0.18 | 0.6908 | NC | NC |
|  | Rec |  | 0.3659 | 0.337 |  | -1.11 | 0.7668 | 0.8341 | 0.5683 |
| rs3768293 | Add |  | 0.2051 | 0.2051 |  | -0.04 | 0.853 | 0.7625 | 0.7625 |
|  | Dom |  | 5 x 10^-6^ | 7 x 10^-6^ |  | 1.24 | 0.0016 | 0.0016 | 0.0018 |
|  | Rec |  | 0.0828 | 0.0834 |  | -0.87 | 0.0061 | 0.0038 | 0.0039 |
| rs6603867 | Add |  | 0.1501 | 0.1505 |  | 0.14 | 0.5547 | 0.5388 | 0.5388 |
|  | Dom |  | 2 x 10^-5^ | 2 x 10^-5^ |  | 1.58 | 2 x 10^-4^ | 1 x 10^-4^ | 1 x 10^-4^ |
|  | Rec |  | 0.2771 | 0.2775 |  | -0.56 | 0.0656 | 0.0682 | 0.0686 |
| rs6678616 | Add |  | 0.2234 | 0.2229 |  | 0.85 | 0.0013 | 0.0019 | 0.0018 |
|  | Dom |  | 0.0215 | 0.0217 |  | 1.24 | 6 x 10^-5^ | 7 x 10^-5^ | 7 x 10^-5^ |
|  | Rec |  | 0.1791 | 2 x 10^-5^ |  | -0.18 | 0.7775 | 0.7215 | 0.7222 |
| rs1472408 | Add |  | 0.2089 | 0.209 |  | 0.07 | 0.7707 | 0.8494 | 0.8494 |
|  | Dom |  | 0.0039 | 0.0042 |  | 1.05 | 0.0119 | 0.012 | 0.0126 |
|  | Rec |  | 0.7184 | 0.7185 |  | -0.48 | 0.1367 | 0.1073 | 0.1081 |
| rs6603883 | Add |  | 0.4119 | 0.4119 |  | 0.10 | 0.6717 | 0.5245 | 0.5245 |
|  | Dom |  | 0.6492 | 0.649 |  | 0.55 | 0.0833 | 0.0546 | 0.0552 |
|  | Rec |  | 0.3489 | 0.3499 |  | -0.71 | 0.1051 | 0.1416 | 0.1437 |
| rs11260822 | Add |  | 0.7543 | 0.7543 |  | -0.13 | 0.5934 | 0.4998 | 0.4998 |
|  | Dom |  | 0.0672 | 0.0684 |  | 0.89 | 0.033 | 0.0402 | 0.0412 |
|  | Rec |  | 0.0757 | 0.0764 |  | -0.77 | 0.0183 | 0.012 | 0.0122 |
| rs904106 | Add |  | 0.5082 | 0.5093 |  | 0.65 | 0.1958 | 0.1174 | 0.1125 |
|  | Dom |  | 0.5082 | 0.5093 |  | 0.45 | 0.3886 | 0.2613 | 0.2583 |
|  | Rec |  | NA | NA |  | 8.31 | 0.0135 | 0.0064 | 0.0105 |
| rs729402 | Add |  | 0.1977 | 0.1976 |  | -0.04 | 0.8669 | 0.7376 | 0.7375 |
|  | Dom |  | 0.0006 | 0.0007 |  | 1.09 | 0.0112 | 0.0152 | 0.0158 |
|  | Rec |  | 0.5044 | 0.5047 |  | -0.66 | 0.0396 | 0.0273 | 0.0277 |

^a^ Three genetic models are additive (Add), dominant (Dom), and recessive (Rec) models.

^b^ INV-LP: asymptotic P value from likelihood ratio for the inverse normal transformed trait with the George-Elston transformation; INV-WP: asymptotic P value from Wald test for the inverse normal transformed trait with the George-Elston transformation; NA: not applicable because of small variance.

^c^ GE-β: regression coefficient after George-Elston transformation; GE-LP: asymptotic P value from likelihood ratio test after George-Elston transformation; NA: not applicable because of small variance.
