# Supplementary material for: EPHA2 Is Associated with Age-Related Cortical Cataract in Mice and Humans
Source: PLoS Genet. 2009 Jul 31;5(7):e1000584. doi: 10.1371/journal.pgen.1000584 (PMC2712078; doi:10.1371/journal.pgen.1000584)
Supplement: Table S4 — Single SNP association using ASSOC in the UKTS family data. (0.14 MB DOC) [file pgen.1000584.s010.doc]

Table S4. Single SNP association using ASSOC in the UKTS family data

| SNP | Model^a^ |  | Severe Cortical^b^ | |  | Cortical^c^ | | | |
| --- | --- | --- | --- | --- | --- | --- | --- | --- | --- |
|  |  |  | INV-LP | INV-WP |  | GE-β | GE-LP | INV-LP | INV-WP |
| rs924201 | Add |  | 0.4141 | 0.4204 |  | 0.66 | 0.0853 | 0.6991 | 0.7008 |
|  | Dom |  | 0.0618 | 0.0641 |  | 4.91 | 0.0010 | 0.0087 | 0.0086 |
|  | Rec |  | 0.0105 | 0.0099 |  | -1.97 | 0.0346 | 0.1458 | 0.1465 |
| rs7548209 | Add |  | 0.0205 | 0.0178 |  | 0.06 | 0.9590 | 0.8089 | 0.8110 |
|  | Dom |  | 3 x 10^-4^ | 3 x 10^-4^ |  | 2.58 | 0.0797 | 0.0524 | 0.0531 |
|  | Rec |  | 0.5904 | 0.5916 |  | -4.89 | 0.0097 | 0.0199 | 0.0191 |
| rs1803527 | Add |  | MS | MS |  | MS | MS | MS | MS |
|  | Dom |  | MS | MS |  | MS | MS | MS | MS |
|  | Rec |  | MS | MS |  | MS | MS | MS | MS |
| rs3754334 | Add |  | 0.0425 | 0.0432 |  | -0.32 | 0.6874 | 0.9020 | 0.9020 |
|  | Dom |  | 1 x 10^-4^ | 1 x 10^-4^ |  | 2.95 | 0.0468 | 0.0255 | 0.0253 |
|  | Rec |  | 0.0804 | 0.0785 |  | -7.52 | 2 x 10^-5^ | 5 x 10^-4^ | 5 x 10^-4^ |
| rs11260721 | Add |  | 0.6206 | 0.621 |  | -0.52 | 0.7475 | 0.6982 | 0.6977 |
|  | Dom |  | 0.4647 | 0.4653 |  | -1.54 | 0.3775 | 0.3766 | 0.3757 |
|  | Rec |  | 0.2803 | 0.2867 |  | 47.33 | 0.0037 | 0.0062 | 0.0087 |
| Ile779Ile | Add |  | 3 x 10^-5^ | 2 x 10^-5^ |  | -4.95 | 0.0911 | 0.1059 | 0.1013 |
|  | Dom |  | 3 x 10^-5^ | 2 x 10^-5^ |  | -4.95 | 0.0911 | 0.1059 | 0.1013 |
|  | Rec |  | NA | NA |  | NA | NA | NA | NA |
| Arg721Gln | Add |  | MS | MS |  | MS | MS | MS | MS |
|  | Dom |  | MS | MS |  | MS | MS | MS | MS |
|  | Rec |  | MS | MS |  | MS | MS | MS | MS |
| rs13375644 | Add |  | 0.1987 | 0.1957 |  | -2.14 | 0.1936 | 0.3080 | 0.3088 |
|  | Dom |  | 0.1987 | 0.1957 |  | -2.14 | 0.1936 | 0.3080 | 0.3088 |
|  | Rec |  | NA | NA |  | NA | NA | NA | NA |
| rs2230597 | Add |  | 0.7401 | 0.7405 |  | -1.05 | 0.3580 | 0.4067 | 0.4058 |
|  | Dom |  | 0.1577 | 0.1575 |  | 2.00 | 0.2690 | 0.2438 | 0.2455 |
|  | Rec |  | 0.0104 | 0.0097 |  | -5.99 | 0.0065 | 0.0017 | 0.0014 |
| Ser277Leu | Add |  | 0.9295 | 0.9296 |  | 12.97 | 0.3664 | 0.0976 | 0.1065 |
|  | Dom |  | 0.9295 | 0.9296 |  | 12.97 | 0.3664 | 0.0976 | 0.1065 |
|  | Rec |  | NA | NA |  | NA | NA | NA | NA |
| rs11260745 | Add |  | 0.318 | 0.3199 |  | -4.99 | 0.0060 | 0.0085 | 0.0084 |
|  | Dom |  | 0.318 | 0.3199 |  | -4.99 | 0.0060 | 0.0085 | 0.0084 |
|  | Rec |  | NA | NA |  | NA | NA | NA | NA |
| rs3768293 | Add |  | 0.0875 | 0.0878 |  | 0.68 | 0.6143 | 0.6224 | 0.6227 |
|  | Dom |  | 0.0040 | 0.0040 |  | 2.94 | 0.0121 | 0.0429 | 0.0432 |
|  | Rec |  | 0.5398 | 0.5388 |  | -4.02 | 0.0453 | 0.0430 | 0.0409 |
| rs6603867 | Add |  | 0.9018 | 0.9623 |  | 0.84 | 0.4169 | 0.5589 | 0.5621 |
|  | Dom |  | 0.6685 | 0.6802 |  | 2.96 | 0.1050 | 0.1614 | 0.1615 |
|  | Rec |  | 0.8781 | 0.9182 |  | -0.12 | 0.9366 | 0.8325 | 0.8329 |
| rs6678616 | Add |  | 0.0214 | 0.0215 |  | 0.41 | 0.7171 | 0.6325 | 0.6339 |
|  | Dom |  | 0.015 | 0.0152 |  | 0.78 | 0.5946 | 0.5245 | 0.5249 |
|  | Rec |  | 0.3237 | 0.3222 |  | -0.22 | 0.9240 | 0.9894 | 0.9894 |
| rs1472408 | Add |  | 0.9406 | 0.8495 |  | 0.92 | 0.3954 | 0.4409 | 0.4434 |
|  | Dom |  | 0.8597 | 0.8037 |  | 1.35 | 0.5105 | 0.5844 | 0.5854 |
|  | Rec |  | 0.9794 | 0.8634 |  | 1.07 | 0.4797 | 0.4997 | 0.5011 |
| rs6603883 | Add |  | 0.8802 | 0.8226 |  | -0.63 | 0.5714 | 0.6352 | 0.6370 |
|  | Dom |  | 0.9992 | 0.865 |  | 0.09 | 0.9515 | 0.9287 | 0.9279 |
|  | Rec |  | 0.7719 | 0.7352 |  | -2.41 | 0.2407 | 0.2891 | 0.2910 |
| rs11260822 | Add |  | 0.9313 | 0.7935 |  | 0.78 | 0.4859 | 0.5474 | 0.5472 |
|  | Dom |  | 0.7102 | 0.7059 |  | 2.47 | 0.2313 | 0.2866 | 0.2841 |
|  | Rec |  | 0.8856 | 0.8748 |  | 0.16 | 0.9177 | 0.9401 | 0.9417 |
| rs904106 | Add |  | 2 x 10^-4^ | 9x 10^-5^ |  | -2.61 | 0.2148 | 0.1782 | 0.1765 |
|  | Dom |  | 6 x10^-4^ | 4 x 10^-4^ |  | -2.21 | 0.3547 | 0.2606 | 0.2593 |
|  | Rec |  | 0.0046 | 0.0038 |  | -8.04 | 0.1673 | 0.2090 | 0.2018 |
| rs729402 | Add |  | 0.9803 | 0.9803 |  | 0.89 | 0.4308 | 0.5014 | 0.5066 |
|  | Dom |  | 0.6858 | 0.6894 |  | 3.12 | 0.1363 | 0.1698 | 0.1711 |
|  | Rec |  | 0.7976 | 0.7976 |  | 0.05 | 0.9737 | 0.9871 | 0.9651 |

^a^ Three genetic models are additive (Add), dominant (Dom), and recessive (Rec) models.

^b^ INV-LP: asymptotic P value from likelihood ratio for the inverse normal transformed trait with the George-Elston transformation; INV-WP: asymptotic P value from Wald test for the inverse normal transformed trait with the George-Elston transformation; NA: not applicable because of small variance; MS: monomorpic SNP

^c^ GE-β: regression coefficient after George-Elston transformation; GE-LP: asymptotic P value from likelihood ratio test after George-Elston transformation; NA: not applicable because of small variance; MS: monomorpic SNP
