# Supplementary material for: EPHA2 Is Associated with Age-Related Cortical Cataract in Mice and Humans
Source: PLoS Genet. 2009 Jul 31;5(7):e1000584. doi: 10.1371/journal.pgen.1000584 (PMC2712078; doi:10.1371/journal.pgen.1000584)
Supplement: Table S5 — Association results using PLINK in the BMES unrelated data. (A) P values from single SNP association under the dominant model. (B) P values from single SNP trend test for the severe cases and controls with age at least 70 years in the BMES. (C) Haplotype association for the binary and quantitative cortical cataract trait in BMES. (0.10 MB DOC) [file pgen.1000584.s011.doc]

Table S5. Association results using PLINK in the BMES unrelated data

(A) P values from single SNP association under the dominant model

| SNP | RA^a^ |  | Severe Cortical^b^ | |  | Cortical^b^ | |
| --- | --- | --- | --- | --- | --- | --- | --- |
|  |  |  | β | P |  | β | P |
| rs924201 | A |  | 0.56 | 0.3603 |  | 0.27 | 0.6453 |
| rs7548209 | C |  | -0.70 | 0.1051 |  | 0.85 | 0.0365 |
| rs1803527 | C |  | NA | NA |  | NA | NA |
| rs3754334 | A |  | -0.90 | 0.0196 |  | 0.67 | 0.6453 |
| rs11260721 | G |  | -0.96 | 0.0818 |  | 0.08 | 0.0365 |
| Ile779Ile | T |  | NA | NA |  | NA | NA |
| Arg721Gln | A |  | NA | NA |  | NA | NA |
| rs13375644 | A |  | -0.81 | 0.2435 |  | -0.60 | 0.3758 |
| rs2230597 | A |  | -0.30 | 0.4575 |  | 0.19 | 0.6388 |
| Ser277Leu | T |  | NA | NA |  | NA | NA |
| rs11260745 | G |  | -0.31 | 0.6274 |  | -0.71 | 0.2805 |
| rs3768293 | G |  | -0.82 | 0.0433 |  | 0.42 | 0.2705 |
| rs6603867 | C |  | 1.16 | 0.0435 |  | -0.63 | 0.2411 |
| rs6678616 | A |  | -0.42 | 0.3788 |  | 0.62 | 0.1520 |
| rs1472408 | T |  | 1.31 | 0.0224 |  | -0.07 | 0.8916 |
| rs6603883 | T |  | -0.49 | 0.2797 |  | 0.27 | 0.5177 |
| rs11260822 | A |  | 1.29 | 0.0298 |  | 0.05 | 0.9310 |
| rs904106 | T |  | -0.73 | 0.3399 |  | 0.36 | 0.5924 |
| rs729402 | A |  | 1.06 | 0.0695 |  | 0.10 | 0.8569 |

^a^ RA: reference alleles in the BMES

^b^ β: estimates from regression models; NA: not applicable because of too small minor allele frequency.

(B) P values from single SNP trend test for the severe cases and controls with age at least 70 years in the BMES.

| SNP | RA^a^ | F_A^b^ | F_U^c^ | P^d^ | OR (95% CI)^e^ |
| --- | --- | --- | --- | --- | --- |
| rs924201 | G | 0.241 | 0.403 | 0.0209 | 0.47 (0.25, 0.9) |
| rs7548209 | C | 0.167 | 0.373 | 0.0027 | 0.34 (0.16, 0.7) |
| rs1803527 | C | 0.019 | 0.032 | 0.5941 | 0.58 (0.07, 4.48) |
| rs3754334 | A | 0.148 | 0.330 | 0.0065 | 0.35 (0.16, 0.77) |
| rs11260721 | G | 0.037 | 0.109 | 0.0992 | 0.32 (0.07, 1.34) |
| Ile779Ile | T | NA | NA | NA | NA |
| Arg721Gln | A | NA | NA | NA | NA |
| rs13375644 | A | 0.019 | 0.063 | 0.1850 | 0.28 (0.04, 2.09) |
| rs2230597 | A | 0.259 | 0.436 | 0.0127 | 0.45 (0.24, 0.85) |
| Ser277Leu | T | NA | NA | NA | NA |
| rs11260745 | G | 0.056 | 0.068 | 0.7317 | 0.81 (0.24, 2.74) |
| rs3768293 | G | 0.259 | 0.434 | 0.0140 | 0.46 (0.24, 0.86) |
| rs6603867 | C | 0.259 | 0.416 | 0.0265 | 0.49 (0.26, 0.93) |
| rs6678616 | A | 0.240 | 0.385 | 0.0452 | 0.50 (0.26, 1.00) |
| rs1472408 | C | 0.296 | 0.437 | 0.0486 | 0.54 (0.29, 1.00) |
| rs6603883 | T | 0.278 | 0.423 | 0.0402 | 0.52 (0.28, 0.98) |
| rs11260822 | G | 0.278 | 0.421 | 0.0438 | 0.53 (0.28, 0.99) |
| rs904106 | T | 0.056 | 0.044 | 0.6885 | 1.29 (0.37, 4.52) |
| rs729402 | G | 0.296 | 0.423 | 0.0737 | 0.57 (0.31, 1.06) |

NA: not applicable because of too small minor allele frequency.

^a^ Reference alleles for case-control severe cortical cataract in the BMES, and bold alleles represent alleles that were different from the original reference alleles. Bold letters indicate alleles that are different from the reference alleles in the other tables.

^b^ Frequency of a reference allele in affected.

^c^ Frequency of a reference allele in unaffected.

^d^ Asymptotic P value using a reference allele from Crochran-Armitage trend test in individuals with age at least 70 years contrasting extreme cases (cortical score $\geq$25%) and clear controls (cortical score < 1%)

^e^ Estimated odds ratio for a reference allele and 95% confidence interval of odds ratio.

(C) Haplotype association for the binary and quantitative cortical cataract trait in BMES.

| SNPs^a^ | Haplotype | Β^b^ | t-statistic^c^ | P^d^ |
| --- | --- | --- | --- | --- |
| rs7548209-rs3754334 | CG | 2.36 | 3.04 | 0.0024 |
| rs7548209-rs3754334 | GG | -0.73 | -2.33 | 0.0204 |
| rs6603867-rs6678616 | CA | 6.57 | 4.93 | 1 x 10^-6^ |

Results with P values < 0.05 are shown.

^a^ Using the five most significant SNPs (rs7548209, rs3754334, rs3768293, rs6603867, and rs6678616) moving windows of different sizes (2 – 5) were applied.

^b^ Regression coefficient.

^c^ Test statistic for an effect size ($\beta$) from ordinary least squares regression using a t-statistic

^d^ Asymptotic significance values for the coefficients.
