# Supplementary material for: EPHA2 Is Associated with Age-Related Cortical Cataract in Mice and Humans
Source: PLoS Genet. 2009 Jul 31;5(7):e1000584. doi: 10.1371/journal.pgen.1000584 (PMC2712078; doi:10.1371/journal.pgen.1000584)
Supplement: Table S6 — Heterogeneity test and meta-analysis using METAL (A to D). Three genetic models (Add: additive, Dom: dominant, and Rec: recessive) were tested for each SNP. QE-P: P values associated with the Q statistic; Zscore: Z-score from meta-analysis; N: the total number of individuals in the meta-analysis; Meta-P: P values from meta-analysis. (A) Heterogeneity test (Q test) and meta-analysis for the quantitative cortical cataract from the BDES and UKTS. (B) Heterogeneity test (Q test) and meta-analysis for severe cortical cataract from the BDES and UKTS. (C) Heterogeneity test (Q test) and meta-analysis from all three datasets (BDES, UKTS, and BMES) under the dominant model. (0.23 MB DOC) [file pgen.1000584.s012.doc]

Table S6. Heterogeneity test and meta-analysis using METAL (A to D). Three genetic models (Add: additive, Dom: dominant, and Rec: recessive) were tested for each SNP. QE-P: P values associated with the Q statistic; Zscore: Z-score from meta-analysis; N: the total number of individuals in the meta-analysis; Meta-P: P values from meta-analysis.

(A) Heterogeneity test (Q test) and meta-analysis for the quantitative cortical cataract from the BDES and UKTS

| SNP | Model |  | Heterogeneity Test | |  | Meta-Analysis | | |
| --- | --- | --- | --- | --- | --- | --- | --- | --- |
|  |  |  | QE | QE-P |  | N | Zscore | Meta-P |
| rs924201 | Add |  | 0.05 | 0.8309 |  | 1304 | 1.42 | 0.1566 |
|  | Dom |  | 0.00 | 1.000 |  | 1304 | 2.26 | 0.0240 |
|  | Rec |  | 2.64 | 0.1043 |  | 1304 | 0.14 | 0.8855 |
| rs7548209 | Add |  | 0.01 | 0.9192 |  | 1325 | -0.79 | 0.4290 |
|  | Dom |  | 4.93 | 0.0264 |  | 1325 | 0.52 | 0.6001 |
|  | Rec |  | 2.30 | 0.1295 |  | 1325 | -1.42 | 0.1561 |
| rs3754334 | Add |  | 0.10 | 0.7470 |  | 1316 | 1.11 | 0.2694 |
|  | Dom |  | 3.15 | 0.0757 |  | 1316 | 1.55 | 0.1205 |
|  | Rec |  | 13.06 | 3 x 10^-4^ |  | 1316 | -0.16 | 0.8727 |
| rs11260721 | Add |  | 1.45 | 0.2279 |  | 1352 | 2.12 | 0.0337 |
|  | Dom |  | 2.90 | 0.0886 |  | 1352 | 1.99 | 0.0464 |
|  | Rec |  | 5.95 | 0.0147 |  | 1352 | 1.36 | 0.1746 |
| Ile779Ile | Add |  | 0.83 | 0.3623 |  | 1337 | -2.10 | 0.0356 |
|  | Dom |  | 0.65 | 0.4188 |  | 1337 | -2.26 | 0.0238 |
| rs13375644 | Add |  | 7.45 | 0.0063 |  | 1359 | -0.03 | 0.9778 |
|  | Dom |  | 6.95 | 0.0084 |  | 1359 | -0.03 | 0.9778 |
| rs2230597 | Add |  | 2.54 | 0.1111 |  | 1315 | 1.87 | 0.0616 |
|  | Dom |  | 0.39 | 0.5334 |  | 1315 | 1.57 | 0.1156 |
|  | Rec |  | 14.84 | 1 x 10^-4^ |  | 1315 | 1.45 | 0.1468 |
| Ser277Leu | Add |  | 5.21 | 0.0225 |  | 1279 | -1.39 | 0.1643 |
|  | Dom |  | 5.21 | 0.0225 |  | 1279 | -1.39 | 0.1643 |
| rs11260745 | Add |  | 5.17 | 0.0230 |  | 1039 | -0.71 | 0.4795 |
| rs3768293 | Add |  | 0.33 | 0.5678 |  | 1340 | 0.45 | 0.6519 |
|  | Dom |  | 0.07 | 0.7861 |  | 1340 | 3.65 | 3 x 10^-4^ |
|  | Rec |  | 1.22 | 0.2696 |  | 1340 | -3.40 | 7 x 10^-4^ |
| rs6603867 | Add |  | 0.08 | 0.7822 |  | 1267 | 0.35 | 0.7266 |
|  | Dom |  | 0.27 | 0.6054 |  | 1267 | 1.77 | 0.0763 |
|  | Rec |  | 0.10 | 0.7571 |  | 1267 | -4.13 | 4 x 10^-5^ |
| rs6678616 | Add |  | 0.85 | 0.3552 |  | 1223 | 3.05 | 0.0023 |
|  | Dom |  | 1.03 | 0.3093 |  | 1223 | 3.90 | 1 x 10^-4^ |
|  | Rec |  | 0.03 | 0.8721 |  | 1223 | -0.34 | 0.7375 |
| rs1472408 | Add |  | 0.39 | 0.5315 |  | 1366 | -0.11 | 0.9144 |
|  | Dom |  | 1.62 | 0.2033 |  | 1366 | 1.24 | 0.2153 |
|  | Rec |  | 0.22 | 0.6396 |  | 1366 | -2.54 | 0.0111 |
| rs6603883 | Add |  | 0.65 | 0.1497 |  | 1335 | 0.42 | 0.6718 |
|  | Dom |  | 0.51 | 0.4744 |  | 1335 | 1.81 | 0.0696 |
|  | Rec |  | 0.08 | 0.7805 |  | 1335 | -1.76 | 0.0779 |
| rs11260822 | Add |  | 0.68 | 0.4107 |  | 1330 | 0.85 | 0.3966 |
|  | Dom |  | 0.05 | 0.8249 |  | 1330 | 2.30 | 0.0215 |
|  | Rec |  | 1.18 | 0.2778 |  | 1330 | -2.31 | 0.0211 |
| rs904106 | Add |  | 3.59 | 0.0583 |  | 1325 | 1.06 | 0.2887 |
|  | Dom |  | 2.20 | 0.1385 |  | 1325 | 0.72 | 0.4712 |
| rs729402 | Add |  | 0.56 | 0.4529 |  | 1341 | 0.06 | 0.9521 |
|  | Dom |  | 0.78 | 0.3767 |  | 1341 | 2.06 | 0.0393 |
|  | Rec |  | 0.11 | 0.7395 |  | 1341 | -2.77 | 0.0057 |

(B) Heterogeneity test (Q test) and meta-analysis for severe cortical cataract from the BDES and UKTS

| SNP | Model |  | Heterogeneity Test | |  | Meta-Analysis | | |
| --- | --- | --- | --- | --- | --- | --- | --- | --- |
|  |  |  | QE | QE-P |  | N | Zscore | Meta-P |
| rs924201 | Add |  | 1.42 | 0.2329 |  | 663 | 2.19 | 0.0282 |
|  | Dom |  | 2.30 | 0.1295 |  | 663 | 4.14 | 4 x 10^-5^ |
|  | Rec |  | 6.67 | 0.0098 |  | 663 | -0.93 | 0.3529 |
| rs7548209 | Add |  | 3.22 | 0.0728 |  | 668 | -4.56 | 5 x 10^-6^ |
|  | Dom |  | 0.33 | 0.5678 |  | 668 | 0.03 | 0.9743 |
|  | Rec |  | 9.27 | 0.0023 |  | 668 | -5.78 | 8 x 10^-9^ |
| rs3754334 | Add |  | 2.90 | 0.0887 |  | 656 | 3.05 | 0.0023 |
|  | Dom |  | 12.49 | 4 x 10^-4^ |  | 656 | 3.70 | 2 x 10^-4^ |
|  | Rec |  | 3.71 | 0.0542 |  | 656 | 0.43 | 0.6678 |
| rs11260721 | Add |  | 0.78 | 0.3786 |  | 682 | 2.83 | 0.0047 |
|  | Dom |  | 1.34 | 0.2462 |  | 682 | 3.06 | 0.0022 |
|  | Rec |  | 1.37 | 0.2419 |  | 682 | -0.31 | 0.7549 |
| Ile779Ile | Add |  | 16.78 | 1 x 10^-4^ |  | 664 | -2.09 | 0.0364 |
|  | Dom |  | 16.45 | 1 x 10^-4^ |  | 664 | -2.34 | 0.0191 |
| rs13375644 | Add |  | 1.25 | 0.2629 |  | 688 | 1.62 | 0.1053 |
|  | Dom |  | 1.10 | 0.2934 |  | 688 | 2.01 | 0.0440 |
| rs2230597 | Add |  | 0.33 | 0.5661 |  | 650 | 1.74 | 0.0812 |
|  | Dom |  | 1.68 | 0.1948 |  | 650 | 1.42 | 0.1561 |
|  | Rec |  | 8.07 | 0.0045 |  | 650 | 1.33 | 0.1835 |
| Ser277Leu | Add |  | 0.11 | 0.7422 |  | 633 | -1.74 | 0.0815 |
|  | Dom |  | 0.11 | 0.7422 |  | 633 | -1.74 | 0.0815 |
| rs11260745 | Add |  | 0.96 | 0.3278 |  | 501 | -0.44 | 0.6587 |
|  | Dom |  | 0.97 | 0.3236 |  | 501 | -0.51 | 0.6134 |
| rs3768293 | Add |  | 2.35 | 0.1256 |  | 668 | 1.86 | 0.0628 |
|  | Dom |  | 4.49 | 0.0340 |  | 668 | 5.34 | 9 x 10^-8^ |
|  | Rec |  | 0.20 | 0.6526 |  | 668 | -1.83 | 0.0673 |
| rs6603867 | Add |  | 0.00 | 0.9529 |  | 655 | -1.29 | 0.1977 |
|  | Dom |  | 0.00 | 0.9819 |  | 655 | 1.00 | 0.3189 |
|  | Rec |  | 0.01 | 0.9115 |  | 655 | -3.97 | 0.0001 |
| rs6678616 | Add |  | 4.47 | 0.0345 |  | 634 | 2.21 | 0.0273 |
|  | Dom |  | 4.49 | 0.0342 |  | 634 | 3.19 | 0.0014 |
|  | Rec |  | 1.36 | 0.2427 |  | 634 | -0.67 | 0.5005 |
| rs1472408 | Add |  | 0.06 | 0.8107 |  | 693 | -1.02 | 0.3094 |
|  | Dom |  | 0.01 | 0.9427 |  | 693 | 0.23 | 0.8158 |
|  | Rec |  | 0.30 | 0.5860 |  | 693 | -2.43 | 0.0153 |
| rs6603883 | Add |  | 0.00 | 1.0000 |  | 679 | -0.83 | 0.4049 |
|  | Dom |  | 0.01 | 0.9141 |  | 679 | -0.48 | 0.6291 |
|  | Rec |  | 0.02 | 0.8991 |  | 679 | -0.98 | 0.3272 |
| rs11260822 | Add |  | 0.02 | 0.8999 |  | 676 | 0.41 | 0.6840 |
|  | Dom |  | 0.02 | 0.8883 |  | 676 | 1.79 | 0.0738 |
|  | Rec |  | 0.01 | 0.9339 |  | 676 | -1.64 | 0.1013 |
| rs904106 | Add |  | 13.13 | 3 x 10^-4^ |  | 653 | -2.17 | 0.0299 |
|  | Dom |  | 10.95 | 9 x 10^-4^ |  | 653 | -2.02 | 0.0436 |
| rs729402 | Add |  | 0.02 | 0.8819 |  | 685 | -1.14 | 0.2533 |
|  | Dom |  | 0.00 | 0.9712 |  | 685 | 0.71 | 0.4750 |
|  | Rec |  | 0.03 | 0.8671 |  | 685 | -3.20 | 0.0014 |

(C) Heterogeneity test (Q test) and meta-analysis from all three datasets (BDES, UKTS, and BMES) under the dominant model

| SNP |  | Severe Cortical | | | |  | Cortical | | | |
| --- | --- | --- | --- | --- | --- | --- | --- | --- | --- | --- |
|  |  | QE-P | N | Zscore | Meta-P |  | QE-P | N | Zscore | Meta-P |
| rs924201 |  | 0.2148 | 951 | 3.96 | 9 x 10^-4^ |  | 0.0785 | 2355 | 1.99 | 0.0469 |
| rs7548209 |  | 0.0018 | 956 | -0.86 | 0.3884 |  | 0.0494 | 2375 | 1.78 | 0.0747 |
| rs3754334 |  | 1 x 10^-4^ | 1086 | 1.40 | 0.1602 |  | 0.0438 | 2723 | 2.37 | 0.0180 |
| rs11260721 |  | 0.0935 | 969 | 1.62 | 0.1056 |  | 0.2849 | 2400 | 1.60 | 0.1095 |
| rs13375644 |  | 0.2687 | 976 | 1.06 | 0.2902 |  | 0.4305 | 2411 | -0.61 | 0.5446 |
| rs2230597 |  | 0.3100 | 1081 | 0.63 | 0.5282 |  | 0.6080 | 2697 | 1.44 | 0.1514 |
| rs3768293 |  | 0.0098 | 1096 | 2.91 | 0.0037 |  | 0.5521 | 2743 | 3.34 | 9 x 10^-4^ |
| rs6603867 |  | 0.1581 | 1083 | 2.04 | 0.0409 |  | 0.0095 | 2672 | 0.37 | 0.7110 |
| rs6678616 |  | 0.0876 | 896 | 2.21 | 0.0271 |  | 0.1646 | 2183 | 3.87 | 1 x 10^-4^ |
| rs1472408 |  | 0.0725 | 981 | 1.43 | 0.1518 |  | 0.9352 | 2417 | 0.84 | 0.4000 |
| rs6603883 |  | 0.5657 | 967 | -1.00 | 0.3199 |  | 0.5599 | 2387 | 1.79 | 0.0741 |
| rs11260822 |  | 0.1017 | 963 | 2.68 | 0.0073 |  | 0.9506 | 2381 | 1.78 | 0.0757 |
| rs904106 |  | 0.0022 | 1078 | -2.17 | 0.0300 |  | 0.3545 | 2734 | 0.89 | 0.3757 |
| rs729402 |  | 0.2260 | 973 | 1.59 | 0.1125 |  | 0.8123 | 2392 | 1.66 | 0.0964 |
